# The Fungal Pathogen *Fusarium graminearum* Can Hydrolyse Tryptamine‐Derived Hydroxy‐Cinnamic Acid Amide Defence Compounds, and Generates Highly Increased Auxin Levels in Wheat via Copper Amine‐Oxidases

**DOI:** 10.1111/mpp.70325

**Published:** 2026-08-19

**Authors:** Thomas Svoboda, Eduardo Beltran, Pia Spörhase, Philipp Fruhmann, Anika Bartholomäus, Gerlinde Wiesenberger, Ulrich Gueldener, Bernhard Seidl, Asja Ceranic, Rudolf Krska, Rainer Schuhmacher, Franz Berthiller, Gerhard Adam

**Affiliations:** ^1^ Institute of Microbial Genetics, Department of Agricultural Sciences BOKU University Vienna Austria; ^2^ Institute of Bioanalytics and Agro‐Metabolomics, Department of Agricultural Sciences BOKU University Vienna Austria; ^3^ Institute of Applied Synthetic Chemistry, Faculty of Technical Chemistry TU Wien Vienna Austria; ^4^ Institute of Genome Oriented Bioinformatics, TUM School of Life Sciences Technical University of Munich Freising Germany; ^5^ Core Facility Bioactive Molecules: Screening and Analysis BOKU University Tulln Austria; ^6^ Institute for Global Food Security, School of Biological Sciences Queens University Belfast Belfast Northern Ireland UK

**Keywords:** Fusarium head blight, hydroxycinnamic acid amides, indole‐3‐acetic acid, plant hormone

## Abstract

The important cereal pathogen *Fusarium graminearum* is not only able to produce trichothecene toxins, like deoxynivalenol, but also the plant hormone auxin (indole‐3‐acetic acid, IAA). Highly elevated levels of IAA and auxin derivatives such as IAA‐glucoside or IAA amino‐acid conjugates were observed in the susceptible wheat cultivar Apogee infected with *F. graminearum*. We report here that the fungal pathogen can hydrolyse tryptamine‐derived hydroxycinnamic acid amides produced in high amounts during plant defence and is able to convert the released tryptamine in high yield into IAA. We investigated the role of copper amine‐oxidase candidate genes, which code for enzymes converting tryptamine into the IAA precursor indole‐3‐acetaldehyde. After consecutive knock‐out of seven copper amine oxidases the resulting septuple‐mutant strain (aoxΔ^7^) had strongly reduced ability to produce auxin from tryptamine in vitro. Virulence of the aoxΔ^7^ mutant was significantly impaired while deoxynivalenol production *in planta* was comparable to the wild type. Our results indicate that *F. graminearum,* formerly presumed to be a toxin‐producing necrotroph, in an early biotrophic phase uses subterfuge by converting abundantly formed plant defence compounds into defence‐suppressing auxin.

## Graphical abstract

*Fusarium graminearum* can cleave tryptamine‐derived hydroxycinnamic acid amides and convert the released tryptamine into auxin, suppressing plant defence responses; aoxΔ^7^—septuple knockout of copper amine oxidases blocks auxin formation.

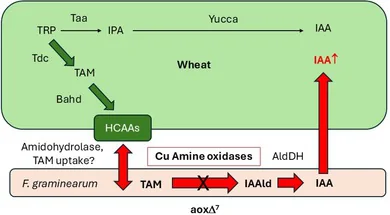

## 1 Introduction

Indole‐3‐acetic acid (IAA, an auxin) is a plant hormone playing a central role in growth‐related processes but also biotic stress responses (Gomes and Scortecci 2021; Waadt et al. 2022). In general, auxin signalling antagonizes salicylic acid (SA)‐mediated defence signalling (Kunkel and Harper 2018), which is effective against biotrophs, and reciprocally, SA inhibits auxin signalling (Du et al. 2013; Tan et al. 2020). Multiple bacteria and fungi can produce auxin, presumably exploiting increased susceptibility caused by elevated auxin signalling. Multiple tryptophan (TRP)‐dependent auxin biosynthetic pathways exist in plants (Cohen and Strader 2024) and in pathogens (Fu et al. 2015) (for overview see Figure S1). While in *Arabidopsis* (*Brassicaceae*) the indole‐3‐acetaldoxime (IAOx) pathway is highly important, the YUCCA pathway is highly relevant in cereals, where TRP is first converted to indole‐3‐pyruvic acid (IPA) by an aminotransferase, and subsequently, in one step, converted into IAA by YUCCA flavin monooxygenases (Zhao et al. 2001). In the indole‐3‐acetamide (IAM) pathway tryptophan is first converted to IAM, which is further metabolized to auxin by indole‐3‐acetamide hydrolase (*iaaH*, *AMI1*). This auxin biosynthetic pathway via tryptophan‐2‐monooxygenase (*iaaM*) and *iaaH* has been reported to be present in many plant‐pathogenic bacteria and also certain members of the *Fusarium fujikuroi* species complex (Niehaus et al. 2016; Tsavkelova et al. 2012). However, this pathway is absent in *Fusarium graminearum*. Many fungi, for example, *Ustilago maydis* and *Neurospora crassa* (Sardar and Kempken 2018), can use transamination of TRP to IPA and use indole‐pyruvate decarboxylase to generate indole acetaldehyde (IAAld). An indole‐pyruvate decarboxylase ortholog is also present in *F. graminearum* (FGSG_03019). Subsequently, aldehyde‐dehydrogenase can convert IAAld into IAA. It has been described that *F. graminearum* is able to produce auxin in vitro from tryptamine (TAM) and indole acetonitrile (IAN) (Luo et al. 2016). Especially in the case of susceptible cultivars, highly elevated levels of auxin have been detected in wheat ears after infection with *F. graminearum* (Wang et al. 2018). Using RNAi‐mediated knockdown, it has been demonstrated that the wheat auxin receptor (*TaTIR1*) mediates susceptibility to *F. graminearum* (Su et al. 2021).

Hydroxycinnamic acid amides (HCAAs), also known as phenolamides (Bassard et al. 2010; Macoy et al. 2015a; Roumani et al. 2021; Xue et al. 2025) are conjugates formed by BAHD acyltransferases (Peng et al. 2016) joining various amines with activated hydroxycinnamic acid derivatives (coumaroyl‐CoA and hydroxylated and methylated derivatives such as feruloyl‐CoA, caffeoyl‐CoA). A total of 74 plant‐derived coumaroyl‐amides were described in a recent review (Berti et al. 2025). HCAAs are typically formed upon biotic stress and are presumed to play an important role in plant defence. In rice, compounds like cinnamoyl‐tryptamine and coumaroyl‐serotonine (COU‐SRT) are induced by infection with the brown spot fungus *Bipolaris maydis* and after UV treatment (Park et al. 2014). Differences exist in the antifungal activity of different compounds and in resistance of fungal isolates. While cinnamoyl‐tryptamine inhibits *Bipolaris oryzae* (IC_50_ about 27 mg/L), no effect of the highest tested concentration could be observed with *Magnaporthe grisea* (*oryzae*) (Park et al. 2014). Yet, the rice genes *TBT1* and *TBT2* encoding tryptamine hydroxycinnamoyl transferases are induced by *M. oryzae* infection. When OsTBT1‐GFP was overexpressed, higher HCAA accumulation and higher resistance against *M. oryzae* were observed (Fang et al. 2022). Serotonin (5‐hydroxy‐TAM) is also used by plants. In bamboo, coumaroyl‐serotonin (COU‐SRT) and feruloyl‐serotonin (FER‐SRT) are induced during infection with fungi causing witches' brooms. Differences in growth inhibition were observed: while *Epichloë bambusae* was unaffected by 100 mg/L COU‐SRT, *Aciculosporum take* was fully inhibited. Yet, both fungi were resistant to 100 mg/L FER‐SRT (Tanaka et al. 2003). While (mostly correlative) evidence suggests a role of HCAAs in plant defence against fungi, bacterial pathogens and herbivores (Xue et al. 2025), their mode of action is hardly known. HCAAs can oxidatively crosslink (Kristensen et al. 2004) either by forming dimers or by reacting with lignin. Furthermore, they are incorporated into cell wall carbohydrates as ethers, crosslinking xylan (Zeiss et al. 2021). Cell wall reinforcement at the site of fungal penetration seems to be an important resistance mechanism. HCAAs have been implicated in *Fusarium* resistance (Gunnaiah and Kushalappa 2014; Karre et al. 2019). The barrier formation in the rachis that limits *Fusarium* spread in the infected ear is suppressed in wheat by the fungal toxin deoxynivalenol (DON) (Jansen et al. 2005). In barley, DON is inactivated by an efficient DON‐inducible UDP‐glucosyltransferase, causing higher *Fusarium* spreading resistance of barley compared to wheat (Bethke et al. 2023).

In this study we focus on the relationship between TAM‐derived defence compounds and auxin biosynthesis under infection conditions. We report that TAM released by hydrolysis is oxidized by amine oxidase (AOX) (Cao et al. 2019; Mano and Nemoto 2012; Sugawara et al. 2009; Zhao 2010) resulting in IAAld, which can be further oxidized to IAA. The goal of our study was to clarify the source of the elevated auxin in *Fusarium*‐infected wheat and to test the relevance of the TAM pathway for *Fusarium* auxin production and virulence by disruption of redundant copper amine oxidase (AOX) candidate genes.

## 2 Results

### 2.1 *Fusarium graminearum* Infection Leads to Highly Elevated Auxin and Auxin‐Metabolites in Apogee Wheat

It has been reported previously (Luo et al. 2016; Shang et al. 2026; Wang et al. 2018) that auxin levels are highly elevated in susceptible wheat cultivars infected by *F. graminearum*. We used liquid chromatography coupled to tandem mass spectrometry (LC–MS/MS) to test the response of the model wheat cultivar Apogee (Mackintosh et al. 2006) used in this study with *F. graminearum* isolate PH‐1 (Cuomo et al. 2007). Upon spray inoculation with conidia, an IAA level of 12.8 mg/kg fresh weight was measured in the pooled ears compared to 0.11 mg/kg in the mock‐treated ears, a roughly 100‐fold difference. In addition to IAA, the conjugate of IAA with aspartic acid (IAA‐Asp) was elevated from below the limit of detection to 18.0 mg/kg. Likewise, a roughly 10‐fold increase of 2‐oxindole‐3‐acetic acid (oxidised auxin) to 1.62 mg/kg was observed in infected wheat. Also, auxin‐glucoside, an unstable substance for which no standard was commercially available and therefore was prepared in‐house (see Experimental Procedures), was present at about 17× higher levels compared to the control. This control experiment confirms that *F. graminearum* infection indeed leads to highly increased auxin levels in Apogee, and that the plant copes with the excess IAA by glycosylation, oxidation (Hayashi et al. 2021), and formation of IAA‐Asp. The latter two reactions are often considered to lead to irreversible auxin‐inactivation (Luo et al. 2023; Rosquete et al. 2012).

### 2.2 *Fusarium graminearum* Can Hydrolyse Coumaroyl‐Tryptamine

Since the first literature report of a microarray experiment on transcriptome changes in barley after *Fusarium* infection, it was recognized that the barley TRP biosynthesis pathway and also TRP‐decarboxylase were upregulated (Boddu et al. 2006). We hypothesized that the main function of TAM might be to serve as precursor for the synthesis of tryptamine‐derived hydroxycinnamic acid amides (HCAAs) (Liu et al. 2022), presumed defence compounds (Doppler et al. 2019; Gunnaiah et al. 2012; Jin and Yoshida 2000; Kushalappa et al. 2016). As the experimental evidence for their antifungal activity is scarce (Jin and Yoshida 2000; Macoy et al. 2015b) we chemically synthesized coumaroyl‐tryptamine (COU‐TAM), and also caffeoyl‐tryptamine (CAF‐TAM) and feruloyl‐tryptamine (FER‐TAM), as well as the analogous derivatives with serotonin (feruloyl‐serotonin, FER‐SRT) and coumaroyl‐serotonin (COU‐SRT) using published procedures (Macoy et al. 2022; Takao et al. 2017).

**FIGURE 1:**
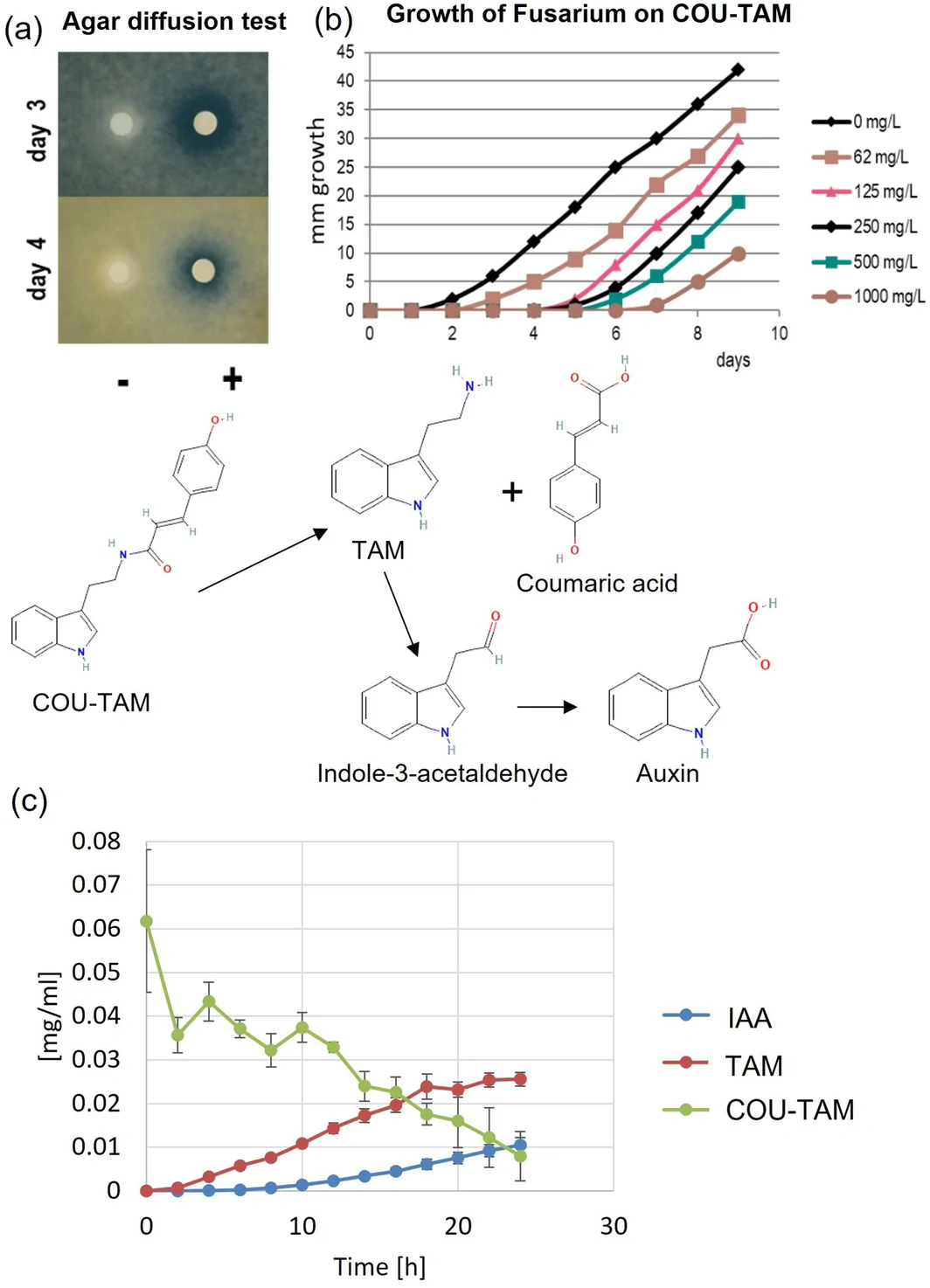
*Fusarium graminearum* COU‐TAM metabolism. (a) Agar diffusion test. On the left side of the plate (−) only solvent was used and on the right side coumaroyl tryptamine (COU‐TAM) was added (+). (b) Concentration dependent growth retardation on COU‐TAM‐containing agar strips. Hypothetical reactions to explain results of (c) COU‐TAM metabolization in liquid medium: Coumaroyl‐tryptamine is cleaved and coumaric acid and tryptamine (TAM) are released. TAM is further converted into indole‐3‐acetaldehyde and further to auxin (IAA).

An agar diffusion test of PH‐1 with COU‐TAM is shown in Figure 1a. While *F. graminearum* is initially inhibited, the zone of inhibition decreases 3–4 days after incubation and is overgrown, indicating that the fungus can eventually overcome the inhibitory effect. Growth inhibition on *Fusarium* minimal medium (FMM) agarose supplemented with different concentrations of COU‐TAM or other compounds ranging from 0 to 1000 mg/L was tested (Spörhase 2015). The latter concentration is above the limit of solubility, and the resulting suspension has a milky appearance. It is evident from Figure 1b that the delay in germination of conidia correlates with the COU‐TAM concentration. However, once adapted, it can grow almost without restraint. Similar growth retardation and subsequent adaptation were also observed with the other compounds (Spörhase 2015). When the fungus was growing at high concentrations of COU‐TAM (and other HCAAs), a clearing zone in the suspension ahead of the fungus was observed.

To test whether inactivation of the compound by a putative secreted amidohydrolase cleaving the conjugate to the corresponding HCA and amine is responsible for this phenomenon, feeding assays in liquid culture were performed. As shown in Figure 1c, the COU‐TAM concentration decreased over time, while TAM increased. After 8 h of incubation, IAA formation was detected, which increased with extended incubation time, while the TAM concentration was about constant between 18 and 24 h and the COU‐TAM levels still dropped. This indicates that the fungus uses TAM released by the cleavage of COU‐TAM as precursor for the formation of IAA.

Auxin formation was observed also with the other HCAAs: 0.5 mM COU‐TAM yielded about 0.37 mM IAA (~75% conversion), while the yield with FER‐TAM and CAF‐TAM was about 35% and 58% respectively. The molar conversion of serotonin derived HCAAs into 5‐hydroxy‐IAA after 80 h was about 43% in the case of COU‐SRT and 81% for FER‐SRT (Spörhase 2015). Pilot experiments indicated that most of the hydrolase activity was not in the cell pellet but in the medium. We therefore set up two experiments where cultures were either induced by COU‐TAM or FER‐TAM or treated with solvent (uninduced). After addition of 0.5 mM to the cultures (and medium without fungus), a slightly milky suspension was formed. In the medium without fungus the turbidity remained, while the fungal cultures were clear at the time of harvest after 24 h. The hydrolase activity of the supernatant with FER‐TAM, which is an abundant HCAA *in planta*, was tested in a time course experiment. The secreted proteins from the FER‐TAM‐induced fungi were able to hydrolyse FER‐TAM rapidly; after 1 h the substrate was already used up and TAM formed (Figure S2). While the observed TAM concentrations were slightly higher than expected (1:1 stoichiometry), the lower measured FER (Figure S2, < 50%) cannot be easily explained by experimental error. In the uninduced samples only slow, time‐dependent, hydrolysis of FER‐TAM was observed. In a control reaction without enzyme, TAM and FER were not observed and FER‐TAM was stable over time. We tested candidate genes selected by a search for ‘amidohydrolase’ in FungiDB (https://fungidb.org/fungidb/app99) or the peptidase database MEROPS (https://www.ebi.ac.uk/merops/), focusing on family M20 and IPR017439 amidohydrolases. However, testing 20 candidates by heterologous expression in *Saccharomyces cerevisiae* did not reveal activity (data not shown).

Next, we set out to test the hypothesis that HCAA‐derived TAM is used as a precursor for auxin synthesis by blocking auxin biosynthesis downstream of the released TAM.

### 2.3 Inactivation of Redundant Amine Oxidase Genes

The first step downstream of TAM is performed by amine oxidases (AOX), either by flavine‐dependent amine oxidases (Pfam: PF01593, EC 1.4.3.4 (Tararina and Allen 2020)) or by copper amine oxidases (Pfam01179, EC 1.4.3.21). These enzymes remove the amino group from primary amines, generating the corresponding aldehyde, ammonium and hydrogen peroxide (KEGG reaction R01853). RNA‐Seq data from a published experiment analysing early time points (Schweiger et al. 2016) indicated that in wheat (CM‐NIL38 containing both resistance genes *Fhb1* and *Qhfs.ifa*‐*5A*) some fungal amine oxidases are expressed as early as 6 h post‐infection, while *TRI5,* which is responsible for trichothecene production, was expressed 24 h post‐inoculation (Table S1 for expression levels and for gene designation). In the genome of *F. graminearum* eight genes are annotated as amine oxidases (Table S1). However, one gene, *AOX5*, is truncated and hardly expressed, so seven genes remained to be disrupted. Single knock‐out strains lacking one amine oxidase, and also double mutants, for example, *aox3 aox4,* showed no reduced production of auxin in vitro (Spörhase 2015).

For the construction of the aoxΔ^7^ (septuple) knock‐out strains, resistance cassettes with positive selection genes (*hph*, *nptII* or *nat1*) fused to the negative selection marker (HSV*tk*) and flanked by two *loxP* sites were used (Twaruschek et al. 2018). HSV*tk* allows negative selection on media supplemented with 5‐fluor‐2‐deoxyuridine, which was used for marker recycling.

Figure S3a shows the scheme of consecutive disruptions. We started with disruption of *AOX3* because, together with *AOX4,* it showed the highest activity in native PAGE with tryptamine upon expression of cDNAs in *S. cerevisiae* (Spörhase 2015). Next, *AOX4* was disrupted in the strain already lacking *AOX3*. Subsequently, protoplasts of a PCR‐confirmed double mutant were treated with recombinant Cre‐protein for marker recycling (Twaruschek et al. 2018). *AOX2* was replaced by the resistance cassette *nat1*‐HSV*tk* (nourseothricin selection) in the double knock‐out strain. The fourth gene that was disrupted in the triple knock‐out strain was *AOX6*. One PCR‐confirmed strain was chosen for pop‐out of the resistance cassettes so that the last three knock‐outs could be performed in a row. We continued with *AOX8* and then tested whether the amount of auxin could be further reduced by disruption of the remaining two amine oxidase candidate genes, *AOX7* and *AOX1*.

After disruption of all seven copper amine oxidases and subsequent marker removal using CRE recombinase, all coding sequences were replaced by a single *loxP* site (Figure S3a). The results of the screening of the aoxΔ^7.1^ knock‐out strain with primer pairs flanking the insertion sites are shown in Figure S3b. All PCR fragments showed the expected size, confirming that all knock‐out steps and Cre‐recombination marker removal events were successful and no chromosomal rearrangements had been induced during recombination. The detailed genotypes of the different strains are summarized in Figure S4.

**FIGURE 2:**
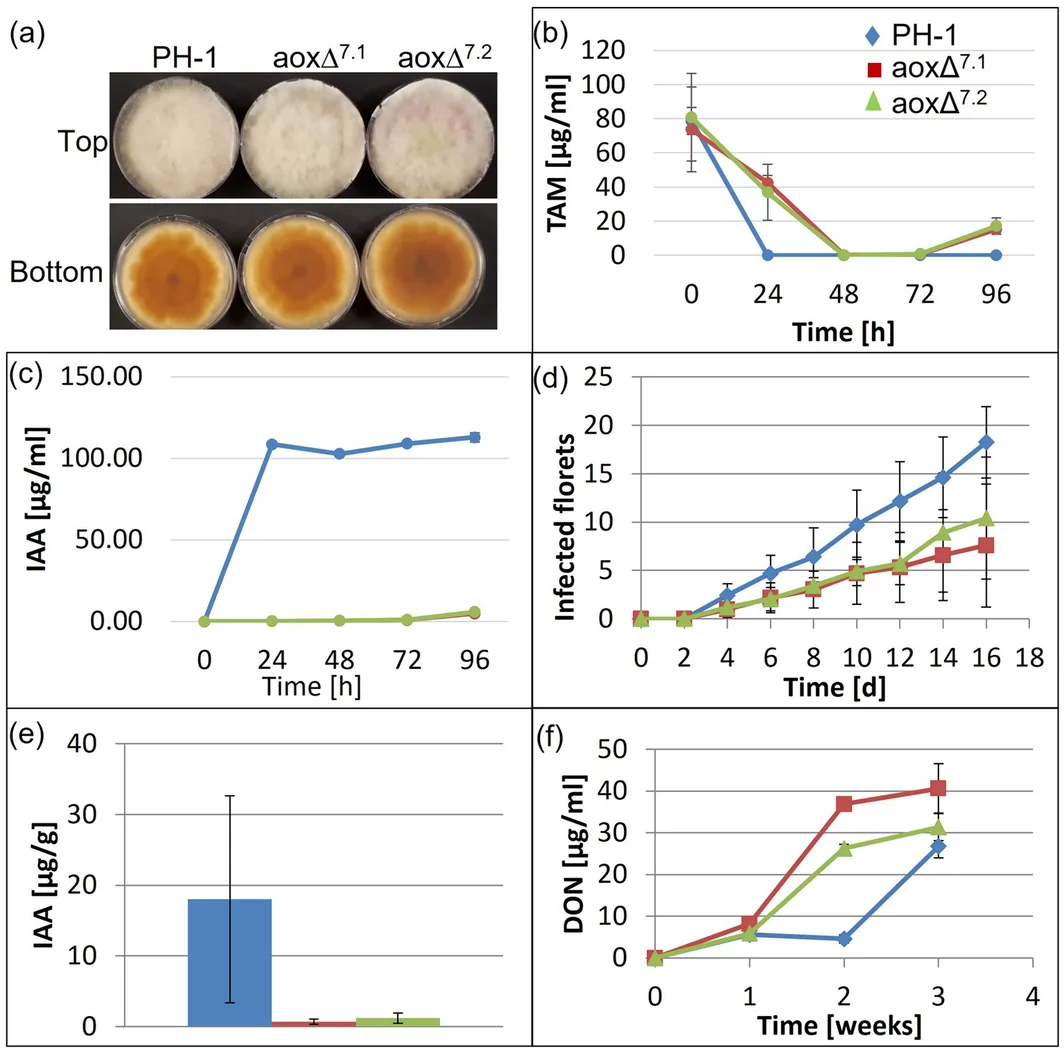
Growth of the aoxΔ^7^ knock‐out strains on potato dextrose agar (view from top and bottom) (a); tests of the aoxΔ7 knock‐out strains compared to the wild‐type strain (PH‐1) for tryptamine (TAM) consumption (0.5 mM TAM added) (b); and indole‐3‐acetic acid (IAA) formation in liquid culture (c); progress of infection on the susceptible wheat cultivar Apogee (d); IAA concentration in infected wheat ears 16 days post‐inoculation (e); deoxynivalenol (DON) formation in liquid culture over time (f).

As shown in Figure 2a, both independent septuple knock‐out strains exhibited comparable growth to the wild type on potato dextrose agar (PDA).

### 2.4 Impaired IAA Production in aox∆^7^ Strains

Once the aoxΔ^7^ knock‐out strains were generated, a TAM feeding assay was performed to determine whether they would be still able to produce auxin in vitro. TAM (Figure 2b) was almost completely converted to IAA (Figure 2c) by the wild‐type strain within 24 h. In the cultures containing the aoxΔ^7^ strains, TAM unexpectedly also disappeared after 48 h; however, no IAA was detected at this time point (Figure 2b). Between 72 and 96 h, about 20% of the added TAM was released again, indicating that most likely a metabolite was formed in a reversible reaction. Low amounts of IAA (about 3% of wild‐type [WT]) were detected in the mutant after 96 h (Figure 2c), which might be due to the presence of either flavin‐dependent amine oxidases from TAM or due to synthesis of IAA by *Fusarium* derived from TRP via other pathways.

### 2.5 Reduced Virulence of aoxΔ^7^ Strains on Wheat

To test if deletion of all seven AOX genes affects virulence, we performed infection assays (point inoculation) with the susceptible wheat cultivar Apogee.

Figure 2d indicates that both aox∆^7^ strains showed a significantly slower spread within wheat ears (*t*‐test: *p* < 0.05) compared to the wild‐type (WT) strain. While the WT strain eventually infected all spikelets, the knock‐out strains advanced only through less than half the ear. The IAA levels in the whole ear were significantly higher in the ears treated with the WT strain, even though there was a high variation among the WT‐treated ears (Figure 2e).

### 2.6 Higher DON Production of aoxΔ^7^ Strains Compared to the Wild‐Type in Liquid Medium

To confirm that the lower virulence of the strains was caused by the strongly impaired auxin production and not due to reduced DON levels, DON production in vitro was determined. A liquid medium (2SM, Lowe et al. 2012) suitable for DON production in vitro was inoculated and incubated for 3 weeks at 20°C in the dark. Samples were taken every week. After 1 week of incubation all strains showed comparable DON levels, and they also reached a similar level at the end point. The amount of DON in the cultures containing the aox∆^7^ strains increased over time, the DON concentration in the WT culture after 2 weeks was significantly lower than in the mutant cultures (Figure 2f). DON production was not impaired in the aox∆^7^ strains; in contrast, DON seemed to be produced somewhat earlier in the mutant in vitro.

**FIGURE 3:**
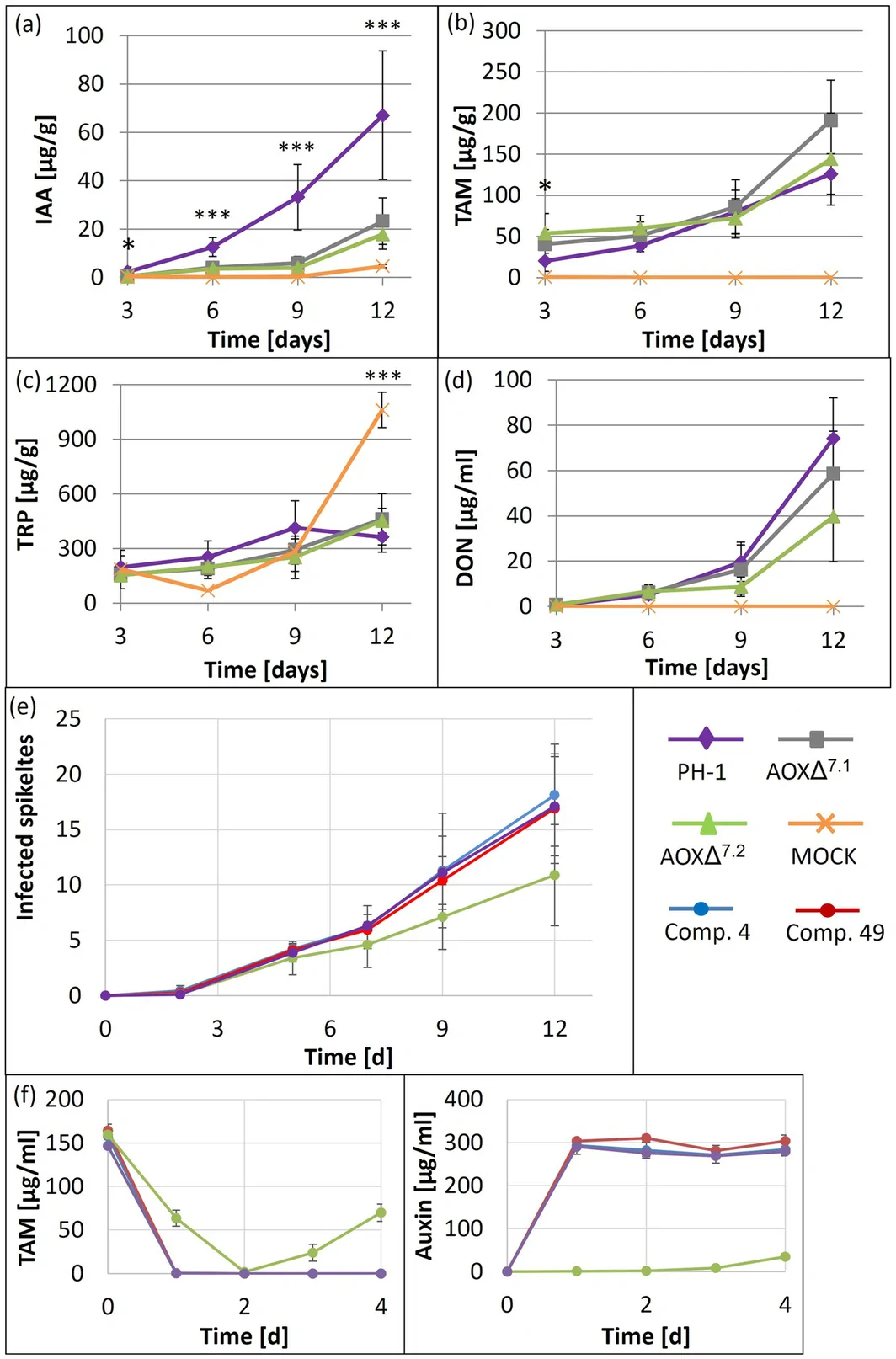
Concentration of indole‐3‐acetic acid (IAA) (a), tryptamine (TAM) (b), tryptophan (TRP) (c) and deoxynivalenol (DON) (d) over time in wheat heads infected with the wild type and the septuple knock‐out strains. The asterisks indicate a significant difference between the wild type and the septuple knock‐out strains. (e) and (f) show the reconstituted virulence in the complementation strains and ability to metabolize TAM to IAA.

Because the aox∆^7^ strains produced only low amounts of IAA compared to the WT strain in culture, we set up an experiment to determine the change of IAA, TAM, TRP and DON levels in infected wheat ears over time (Figure 3a–e). In the ears treated with the WT strain, the IAA levels (Figure 3a) were significantly higher than in those inoculated with the septuple knock‐out strains (*p* < 0.05). In contrast, TAM levels were only significantly higher in the ears infected with the knock‐out strains at the early infection time point 3 days post‐inoculation (dpi). After 6 and 9 dpi, the TAM levels were not significantly elevated, indicating that potentially, like in culture, TAM is metabolized into other still unknown compounds in the mutant *in planta*.

The mock‐inoculated ears showed significantly lower TRP levels at 6 dpi, while a strong increase between 9 and 12 dpi led to significantly higher TRP levels compared to the *Fusarium*‐treated ears (Figure 3c). The DON levels were comparable for WT and mutant‐infected ears until 6 dpi. While aoxΔ^7.1^ showed slightly lower DON levels compared to the WT strain, aoxΔ^7.2^‐infected heads exhibited significantly lower DON levels at 9 and 12 dpi (*p* < 0.01). We had already shown that the aox∆^7^ strains show lower virulence and thus led to a lower number of colonized spikelets per ear compared to the WT strain.

### 2.7 Restoration of Virulence in the Complemented Mutants

To test whether the phenotypes of the mutant are indeed caused by loss of amine oxidase activity, we generated complementation strains by in‐locus reintroduction of *AOX4* into the aox∆^7.2^ mutant. The results of the tests of the complementation strains are shown in Figure 3e,f. In both complemented transformants, rapid TAM metabolization and IAA production in vitro was observed, and the virulence *in planta* was restored to the WT level.

## 3 Discussion

**FIGURE 4:**
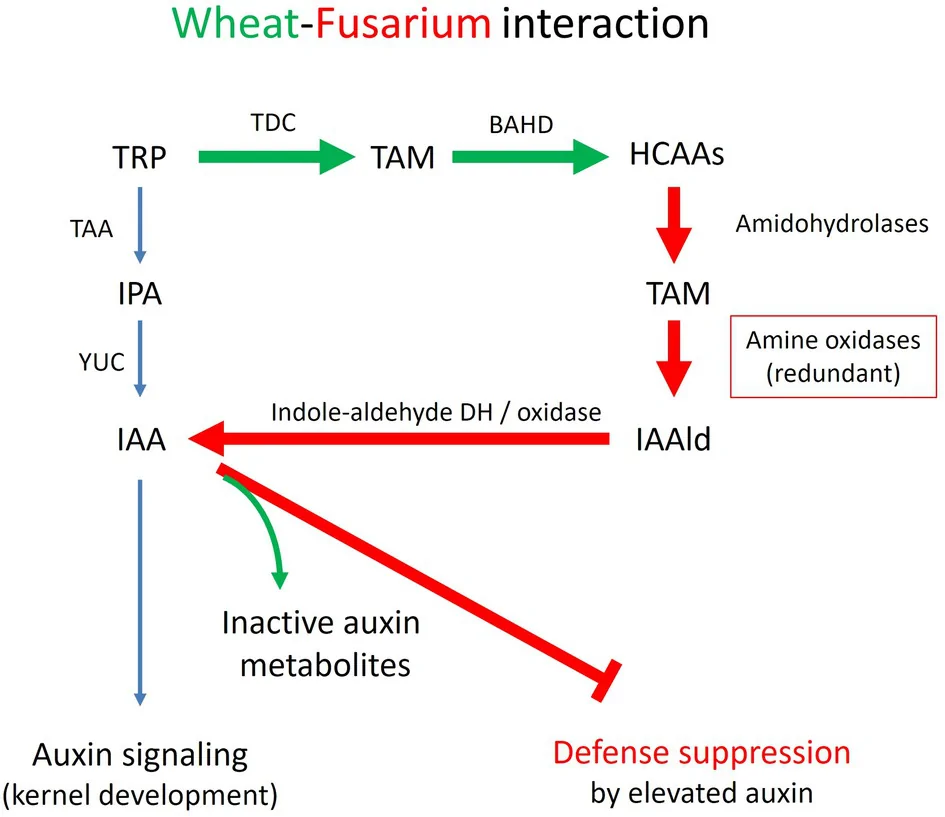
The main auxin biosynthesis pathway required for normal grain development without infection (blue arrows) proceeds mainly via transamination (TAA: tryptophan aminotransferase) and YUCCA flavin monooxygenases converting indole pyruvic acid (IPA) into indole‐3‐acetic acid (IAA). During *Fusarium* infection plant defence (green arrow) causes upregulation of tryptophan (TRP) biosynthesis and formation of tryptamine (TAM) by tryptophan decarboxylase (TDC). TAM is used for synthesis of hydroxycinnamic acid amides (HCAAs) by BAHD N‐acyltransferases. *Fusarium graminearum* uses uncharacterized amidohydrolases as virulence factors (red arrows) that can release TAM from HCAAs. Then redundant copper amine oxidases (AOX) convert indole acetaldehyde (IAAld) into IAA via aldehyde dehydrogenase (DH) or oxidase. Elevated IAA suppresses plant defence, but infected plants can partly counteract by inactivating IAA by conjugation or oxidation.

*Fusarium graminearum* was previously often considered to be a necrotroph (Liao et al. 2022), which—in oversimplification—is assumed to produce trichothecene toxins to kill the host plant and live on the dead tissue. Yet, there is increasing evidence that *F. graminearum* also has an early asymptomatic biotrophic phase (Brown et al. 2017). Our results indicate that IAA generated early in the interaction is relevant for virulence. We envision a scenario (outlined in Figure 4) in which the plants recognize the presence of the invading pathogen and start producing HCAAs, which have been suggested previously to have an impact on the resistance of wheat towards *F. graminearum* (Doppler et al. 2019; Gunnaiah et al. 2012). At present a clear picture about the timing of formation and quantitative spatial distribution (in kernels and rachis) of the different HCAAs during *Fusarium* infection progressing from spikelet to spikelet is missing—such studies are demanding and limited by the lack of commercially available standards.

We obtained evidence that COU‐TAM is inhibiting fungal growth (Figure 1), but that the pathogen can hydrolyse HCAAs, presumably by secreted amidohydrolases. The antifungal activity of the COU‐TAM hydrolysis product, TAM, which is converted to IAA by *Fusarium*, and coumaric acid, is lower than that of the parental compound (Sevinç et al. 2025). *F. graminearum* can use TAM as sole carbon and nitrogen source (Spörhase 2015). TAM becomes toxic in agar medium at concentrations above 125 mg/L (Spörhase 2015), which is in the highest range observed *in planta* under the conditions tested here. TAM is converted into the plant growth hormone IAA that at much lower concentrations is hormonally active and presumed to suppress defence. IAA had been reported to be inhibitory for growth of *F. graminearum* at high concentrations (0.1–1 mM, corresponding to app. 17.5–175 mg/L) in vitro (Luo et al. 2016). The wave of high auxin can presumably diffuse faster than the fungal infection front moves, thereby conditioning a state of susceptibility ahead of the fungus. The high levels of auxin rapidly induce transcripts of plant enzymes involved in auxin inactivation by glycosylation, amino‐acid conjugation and oxidation to return to IAA homeostasis. However, the production of active conjugation enzymes might be blocked or delayed by the protein synthesis inhibitor DON.

One would expect that when DON production is unchanged, the DON‐levels in ears infected by the aox∆^7^ mutant should be also about half when only half of the ear is colonized. However, DON concentrations were not significantly different in ears infected with the aoxΔ^7^ mutant. In contrast to other amines like putrescine and agmatine, which are also converted into HCAAs by wheat, TAM has only a small stimulatory effect in DON production in vitro (Gardiner et al. 2010; Luo et al. 2016). However, loss of all amine oxidases might lead to higher concentrations of other amines, triggering higher DON production *in planta*. Amine oxidases may have different substrate specificities and physiological roles potentially relevant for virulence. The restoration of virulence by reintroduction of a single AOX gene (encoding an enzyme active with TAM) into the aox∆^7^ is evidence against an indirect effect unrelated to auxin.

The loss of the amine oxidases led to lower virulence, potentially because the mutants cannot elevate auxin levels high enough to antagonize SA‐mediated defence (Hao et al. 2018; Makandar et al. 2012). In future work such a postulated effect on SA should be experimentally tested. The situation is complicated by the fact that SA is also targeted directly by *F. graminearum* effector proteins inactivating SA (Qi et al. 2019). An open question is how widespread the hijacking of plant auxin signalling by fungal pathogens via amine oxidase‐mediated auxin production from TAM is. Copper amine oxidases are present in many fungal genomes, so the ability to hydrolyse the HCAAs and create the substrate may be decisive and the actual virulence factor—a topic for future research. According to Lowe et al. (2015) the *F. graminearum* genome contains nearly 400 predicted peptidases, with about 28% metallopeptidases including many predicted secreted proteins. A further complication is that this study revealed a high frequency of non‐canonical secretion. Activity guided fractionation and proteomics should allow identification of the relevant enzymes cleaving FER‐TAM (and other HCAAs). In the experiments with concentrated spent medium (containing secreted proteins) TAM was formed from FER‐TAM at slightly higher levels than expected (1:1 M ratio due to hydrolysis), which ranged within the experimental error. However, the FER levels measured were too low for such an explanation (Figure S2). FER, a reported inhibitor of DON biosynthesis (Boutigny et al. 2009), is known to be fairly unstable (Wang et al. 2011). It has been reported that FER can undergo multiple enzymatic reactions, including peroxidase‐mediated dimerization and polymerization (for review see: Rosazza et al. 1995). Several *Fusarium* species, including *F. graminearum*, have been reported to degrade ferulic acid by decarboxylation (Rosazza et al. 1995). Further research is necessary to determine the metabolic fate of ferulic acid in the enzyme extract.

Interestingly, several of the *F. graminearum* AOX genes (particularly *AOX4*) are highly induced in the living host (Boedi et al. 2016) compared to expression in liquid nitrogen‐inactivated wheat (which is unable to perform a defence response but serves as substrate for saprophytic growth with the same nutrient composition). This indicates that these genes respond to active plant metabolism. The copper amine oxidases are (based on bioinformatics tools) peroxisomal, so that export of IAA is necessary to reach the host. Recently, in *F. graminearum* a putative auxin efflux carrier (FgAec1, a PIN‐like protein) localized in the plasma membrane was described (Hu et al. 2025). Disruption of this gene led to reduced virulence similar to our results with the inactivation of AOX genes.

In *Arabidopsis* infected with *F. oxysporum*, IAA is elevated (Kidd et al. 2011). Yet, *Arabidopsis* lacks tryptophan decarboxylase (Back 2021) and no relevant tryptamine levels have been detected (Kanjanaphachoat et al. 2012), so different mechanisms from the one described here for wheat seem to be operating. Potentially, the fungus might utilize other precursors for defence compounds (e.g., IAN). However, there is increasing evidence that several effector proteins of fungi target the TOPLESS‐related corepressors to trigger auxin signalling in the host (Aalders et al. 2024; Bindics et al. 2022; Navarrete et al. 2022).

In the aoxΔ^7^ mutant residual auxin production was observed in vitro after an extended time, and likewise *in planta* higher auxin levels were detected in the aoxΔ^7^‐infected wheat compared to mock‐treated plants (Figure 3a). In principle, it cannot be excluded that the elevated auxin is due to plant biosynthesis. A more likely scenario is that TAM, still present due to hydrolysis of HCAAs, can be converted by the fungus into auxin in pathways other than those involving copper amine‐oxidases. Potentially, flavine‐dependent amine oxidases might be able to synthesize auxin, or the fungus might synthesize TRP itself or tap into the plant‐derived TRP pool and convert it to auxin in other ways. For instance, YUCCA‐like genes seemingly exist in *Fusarium* but are still uncharacterized (e.g., FGSG_03915, annotated in FungiDB [FGRAMPH1_01G14025] as flavin‐containing monooxygenase yucca10). In a recent review (Shang et al. 2026) several hypotheses on the origin of the elevated auxin in *Fusarium*‐infected wheat have been proposed, some rather questionable such as the conversion of TAM into IAAld via the intermediate *N*‐hydroxyl‐tryptamine (see also: Tivendale et al. (2010)).

The ability of plants to cope with the highly elevated auxin levels triggered by the fungus seems to correlate with resistance (Wang et al. 2018). Processes like glycosylation, oxidation and amino‐acid conjugation of auxin might therefore be relevant for resistance breeding. Another possibility for plants to counteract manipulated auxin levels might be desensitization of auxin signalling components, like for example the main auxin receptor TIR (Su et al. 2021), by RNA interference with microRNAs (Luo et al. 2022). In previous studies, multiple genes implicated in auxin responses have been found to be downregulated in infected wheat (Wang et al. 2018). External application of auxin might be tricky and highly dependent on the timing, making it difficult to distinguish primary and secondary effects. In case of *Fusarium culmorum* and wheat, it has been reported that pretreatment with IAA increased resistance of wheat (Petti et al. 2012), while exogenous auxin triggered susceptibility (Wang et al. 2018).

Auxin is highly relevant for developmental processes leading to embryo development and grain filling (Song et al. 2024). In addition to direct interference with plant defence, elevated auxin levels after *Fusarium* infection might also cause disturbance of the developmental process, leading to formation of the typical shrivelled kernels due to early termination of grain filling by a prematurely triggered succession of auxin by ethylene and abscisic acid (ABA). ABA and derivatives (abscisic aldehyde, 8‐OH‐ABA, ABA‐glucose ester) have been found at increased levels in *Fusarium‐*infected ears (Gunnaiah et al. 2012; Wang et al. 2018). Achieving the right balance (Denancé et al. 2013) between development, grain yield and *Fusarium* resistance remains a difficult task for breeders. Insights from this work can hopefully aid the goal to reduce mycotoxin contamination of grain.

## 4 Experimental Procedures

### 4.1 Chemicals and Reference Substances

If not stated otherwise chemicals were obtained from Sigma‐Aldrich. 5‐hydroxyindole 3‐acetic acid (5OH‐IAA) and oxidized auxin (oxindole‐3‐acetic acid, OxIAA) were from OlChemIm. Indole‐3‐acetyl‐1‐glucosyl ester (IAA‐Glc), which was not commercially available, was generated using an IAA‐treated *S. cerevisiae* expressing the glucosyltransferase UGT84B1 from *Arabidopsis thaliana* (Kovalsky Paris 2013). The unstable compound was isolated by preparative HPLC and was used as a qualitative standard only.

### 4.2 Spray Inoculation Pilot Experiment

Four puffs from a spray bottle (each 130 μL of suspension with 10^5^ spores/mL) were applied per wheat ear from four directions. The ears were covered with plastic bags for 2 days. At 10 dpi four ears were harvested, pooled and ground in liquid nitrogen with mortar and pestle for extraction.

### 4.3 Agar Diffusion Test

To test the growth inhibition by HCAAs in the agar diffusion test, 1 mL water containing 10^5^ conidia of the *F. graminearum* was gently mixed with 12 mL FMM‐agarose (1% agarose at 45°C) and poured into Petri dishes. 2 mg of concentrated COU‐TAM from a stock in acetone was applied to a 6‐mm blank antibiotic disc (Rotilabo KA07.1) and the solvent was evaporated. The disk with the HCAA was then placed on the solidified agar using sterile tweezers.

### 4.4 Growth on HCAA Containing Agar Strips

To save material the growth tests were performed on agar strips prepared on a microscope slide (76 × 26 × 1 mm). The slides were wiped with ethanol, and an 8‐mm wide 0.75‐mm thick teflon spacer from a minigel‐apparatus (Bio‐Rad) was placed in the middle. Two glass slides were placed left and right and one on top, and the setup was fixed with foldback clips. The spacer was removed and one open side was sealed with autoclave tape. The setup was wrapped in aluminium foil and incubated overnight at 65°C. In an Eppendorf tube 590 μL of molten FMM medium containing 1.6% agarose was mixed with 10 μL of HCAA stock (60 mg/mL in dimethyl sulphoxide [DMSO] or 1:2 dilutions thereof, resulting in a final DMSO concentration of 1.7% and concentrations of HCAAs starting at 1000 mg/L). The mix was filled into the space between the glass sides. After removing the top and side glass slides the agarose strips were carefully transferred into 9‐cm Petri dishes by inverting the slides with the agarose strips into one dish each and subsequently lifting the glass slide and leaving the agarose strips sticking to the bottom of the Petri dishes. One end of each strip was inoculated with 5 μL *Fusarium* conidia suspension (containing 1000 spores). The start and growth front were marked daily in the Petri dish with a waterproof pen. The Petri dishes were incubated in a sealed box with wet filter papers to avoid drying out of the agar strips.

### 4.5 Preliminary Investigation of Amidohydrolase Activity

The hydrolysis of FER‐TAM by *F. graminearum* PH‐1 was tested in two independent experiments. Erlenmeyer flasks containing 20 mL of FMM medium were inoculated with 3 × 10^5^ conidiospores and shaken at 20°C and 180 rpm for 90 h. To the dense cultures either 200 μL inducer stock solutions (C—COU‐TAM, F—FER‐TAM) or DMSO (S—solvent control, uninduced) were added. Fresh stocks of COU‐TAM and FER‐TAM (50 mM in DMSO) were prepared, resulting in a final concentration of 0.5 mM in the medium. After 24 h of induction the mycelium was separated from the medium using a Büchner funnel and 70 mm filter paper disks (MN618 G, Macherey‐Nagel, Thermo Fischer Scientific), and 14 mL centrifuged supernatants were loaded onto ultrafiltration spin columns (Amicon Ultra‐15, PLGC Ultracel‐PL Membrane, 10 kDa cut‐off). After centrifugation for 30 min at 3220*g* (Eppendorf centrifuge 5810R with swingout rotor, 4°C) to the remaining 250 μL of concentrated supernatant 1 mL of 1× reaction buffer (50 mM potassium phosphate pH 7, 25 mM NaCl, 2 mM MgSO_4_) was added to remove potentially still present TAM and COU/FER from the induction phase. This was repeated three times. The remaining concentrated secreted enzymes in the supernatant were transferred to Eppendorf tubes stored on ice overnight. On the next day enzymatic reactions were set up in triplicates consisting of 50 μL concentrated supernatant and 450 μL substrate mix added to start the reaction. The final reactions contained 1× reaction buffer, 1% DMSO (to ensure solubility of the substrates) and 125 μM of FER‐TAM. After vortexing 25 μL samples were removed immediately (*t* = 0), and stopped by addition of 200 μL methanol. The reactions were incubated at 30°C and further samples were removed and stopped after 1, 2, 3 and 4 h. To the samples (stored at −20°C) 175 μL ice cold water was added and the mix centrifuged at 4°C and 21,130 *g* for 10 min (Eppendorf centrifuge 5424R). From the supernatant of the protein pellet 380 μL were removed and transferred to new tubes. The samples were frozen at −20°C, thawed, vortexed and centrifuged again. Subsequently 350 μL was transferred to HPLC vials with inserts and crimped. Ferulic acid (ABCR) with a purity of 99% was used to prepare a calibration series in 50% methanol. The samples were analysed using a HPLC system (Vanquish Flex) coupled to an Orbitrap QExactive HF mass spectrometer (Thermo Scientific) equipped with a heated electrospray ionization source. Chromatographic separation was performed as described (Doppler et al. 2022). The following HESI settings were employed: Spray voltage in positive ionization mode 3.6 kV, in negative ionization mode 3.4 kV. Sheath gas 55, aux gas 5 and sweep gas 1 (all arbitrary units). *R* = 120,000 @ 200 *m*/*z*, AGC Target 1e5 and maximum injection time 264 ms.

### 4.6 Plasmid Constructs for Generation of Knock‐Out Strains

For preparation of the disruption constructs approximately 500 bp of upstream and downstream regions flanking the respective genes were PCR amplified using specific primer pairs (containing the required restriction sites for cloning) and PH‐1 genomic DNA as a template and separately cloned into the parental vectors. Primers, plasmids and the names of the resulting plasmids are shown in Table 1, the sequences of the primers are listed in Table 2. Transformations and marker recycling were performed as described by Twaruschek et al. (2018). Screening for transformants with the correct integration events was performed by PCR using the primers described in Table 3. The primers were used according to the scheme previously described (Ćeranić et al. 2021). A detailed description of the events leading to the aoxΔ^7^ septuple construct is outlined in Figure S4.

**TABLE 1:** Cloning strategy of the flanking regions of *AOX* candidate genes. The vectors used in this experiment are described in Twaruschek et al. (2018).

Gene	Name	Region	Cloning sites	Primer pairs	Parental vector	Resulting construct
FGSG_15961	*AOX1*	5′	BcuI/SfiI	2581 + 2582	pKT247 (natI)	pASB9
		3′	HindIII/SalI	2583 + 2584		pASB19
FGSG_03278	*AOX2*	5′	BcuI/SfiI	2827 + 2828	pKT247 (natI)	pPS17
		3′	HindIII/SalI	2829 + 2830		pPS18
FGSG_12129	*AOX3*	5′	BcuI/SfiI	2779 + 2780	pASB43 (hph)	pHE19
		3′	HindIII/SalI	2781 + 2782		pHE27
FGSG_02279	*AOX4*	5′	BcuI/SfiI	2783 + 2784	pASB43 (hph)	pHE49
		3′	SplI/SalI	2785 + 2786		
FGSG_10587	*AOX6*	5′	BcuI/SfiI	3003 + 3004	pKT245 (nptII)	pPS23
		3′	HindIII/SalI	3005 + 3006		pPS24
FGSG_10677	*AOX7*	5′	BcuI/SfiI	3089 + 3090	pKT245 (nptII)	pPS25
		3′	HindIII/SalI	3091 + 3092		pPS26
FGSG_13604	*AOX8*	5′	BcuI/SfiI	2831 + 2832	pKT248 (hph)	pPS56
		3′	HindIII/SalI	2833 + 2834		

**TABLE 2:** Primers used for cloning of the flanking regions for the knock‐out constructs.

Primer #	Primer designation	Sequence
2581	FGSG_15961Dup_SfiI‐fw	TAATGGCCGCATAGGCCAAGTGACAGAGCCTCCT
2582	FGSG_15961Dup_SpeI‐rv	AAACTAGTGATGGTTGTTTTCTGGACTA
2583	FGSG_15961Ddown_SalI‐fw	AATGTCGACGGTAAATGAGTTTGATATCGG
2584	FGSG_15961Ddown_HindIIIrv	ACAAGCTTACTAGCATTAACTGTTGGTTG
2779	FGSG_12129Dup_SfiI‐fw	TAATGGCCGCATAGGCCATTCTAATAGTCAACTGTGTG
2780	FGSG_12129Dup_Spe‐rv	TTAACTAGTGATTGCTGTGTTGAAAAGTC
2781	FGSG_12129Ddown_SalI‐fw	ATTGTCGACAAATCTAGATTGTATGAGTTTGG
2782	FGSG_12129Ddown_HindIII‐rv	TATAAGCTTGCTCTGACCCGAGCATTTTC
2783	FGSG_02279Dup_SfiI‐fw	TAATGGCCGCATAGGCCAGGTTCTAACTCGTAACGTT
2784	FGSG_02279Dup_Spe‐rv	TTAACTAGTGCTGGATAAGACAATAGGTA
2785	FGSG_02279Ddown_SalI‐fw	ATTGTCGACATTATCAGTTTGTAAAAATATGA
2786	FGSG_02279Ddown_SplI‐rv	TATCGTACGCGACGATCCAGATTCATC
2827	Aox2‐5′UTR‐fw‐sfiI	TAATGGCCGCATAGGCCTCTCGAGTAGAGTCTTTGCTA
2828	Aox2‐5′UTR‐rv‐speI	TATACTAGTCTGGAGCGTGATCAAGATGA
2829	Aox2‐3′UTR‐fw‐SalI	CAAGTCGACCCTCAACCCCGTCAATGGA
2830	Aox2‐3′UTR‐rv‐HindIII	TATAAGCTTCGACTAATAATTGTATATTGAGAT
2831	Aox8‐5′UTR‐fw‐sfiI	TAATGGCCGCATAGGCCATAAAATGACCTACTGTCTCT
2832	Aox8‐5′UTR‐rv‐SpeI	TATACTAGTCGCTTATGCAGAAGAGACGA
2833	Aox8‐3′UTR‐fw‐SalI	CAAGTCGACTCTACCAACACCCGCAAAGT
2834	Aox8‐3′UTR‐rv‐HindIII	TATAAGCTTAGTGAGGCCAAAATTACCCAT
3003	Aox6 disruption SfiI	TAATGGCCGCATAGGCCATCTTCGGTCTTTCGGACAG
3004	Aox6 disruption SpeI	TATACTAGTTCGCTTTCTGTCGTATCTCG
3005	Aox6 disruption SalI	CAAGTCGACTTTTCGCATGGTGTTGGATAG
3006	Aox6 disruption HindIII	TATAAGCTTCTAACGTTTCATTCAGTCTTTC
3089	Aox7 disruption SfiI	TAATGGCCGCATAGGCCTAACCCACTCCATTCACTATC
3090	Aox7 disruption SpeI	TATACTAGTGCGCGGAAATGTCGGCAC
3091	Aox7 disruption HindIII	TATAAGCTTTTAACAAGAAAATAAATGAGAGTC
3092	Aox7 disruption SalI	CAAGTCGACTAATACTAAGTGTCACGGCTC

**TABLE 3:** Screening primers used to confirm the knock‐outs.

Primer #	Primer designation	Sequence
1046	NPTII Trafo fw	cctgtccggtgccctgaatga
1047	NPTII Trafo rv	acacccagccggccacagtc
2579	FGSG_15961 + 1‐fwd	ATGATTCCAGACACCTCTTCTAT
2630	Amds_rev	ACACCTGCCGTGTCAGCC
2783	FGSG_02279Dup_SfiI‐fw	TAATGGCCGCATAGGCCAGGTTCTAACTCGTAACGTT
2786	FGSG_02279Ddown_SplI‐rv	TATCGTACGCGACGATCCAGATTCATC
2801	Hyg3_fw	AGAAGTACTCGCCGATAGTG
2802	FGSG_02279‐529_fw (A)	CGAAACTTGTCGGCTGTGG
2803	FGSG_02279 + 466_rv	GGTGGGAGCTTGATCTTGG
2804	FGSG_02279 + 1807_fw (B)	ACAGCGTGCAAGCCAAACG
2806	FGSG_02279 + 2724_rv	ACGATGATCAAACGTGATTCG
2807	FGSG_12129‐555fw (A)	TGTTTCTGGCATGTTCCATAG
2808	FGSG_12129 + 242rv	TCGACAAGTGCATCATGGAC
2809	FGSG_12129 + 1781_fw	ACCGCGACGATGAACTCTG
2810	FGSG_12129 + 2695_rv (B)	GACTAAGGTAGCGGCTTGC
2864	Aox8_confirmation‐A_fw	TATCCCAGCAGAAAAGTTGACC
2865	Aox8_confirmation‐B_rv	CGGGTTATGAGTGAGGCCA
2866	Aox8_confirmation‐C_rv	TTCCCTCGTGTCAGTAATCATG
2867	Aox8_confirmation‐D_fw	GGATGCGATTGCCTTGGAG
2928	Aox2 Confirmation A	TGGGGAGTCATGATGCGATA
2929	Aox2 Confirmation B	AATCGCTTACAATTCTGCGAC
2930	Aox2 Confirmation C	GAACATAGTACGCGACAAAGA
2931	Aox2 Confirmation D	TGACTACGATCTCAGCCGG
3007	Aox6 Confirmation A	TCTATAGCTAACCGATTGGAC
3008	Aox6 Confirmation B	GATGTGGTATGTCTCGTGTTA
3009	Aox6 Confirmation C	ACAAGAATGGGAGAGTATCAC
3010	Aox6 Confirmation D	ACACAGGTGATCTTCCCAAC
3128	Confirmation A Aox7	CTGCTACAGCACCAATCAAAA
3129	Confirmation B Aox7	GAGGAGCTCAGGGAACAAG
3130	Confirmation C Aox7	AGCAGACGATTAATCGCATAG
3131	Confirmation D Aox7	TTTGGAACGAAGCAGCCTAC
3579	Hsvtk‐nptII F	GTAGACCGCAAATGAGCAAC
3580	Hsvtk‐ E	GCCACAGCAGCCACGACA
3952	F: Nat1	ATCTAAAGATCGATTCGGCAG
3987	E 97 bp‐HSVtk	AGGCTCACGAGTCAGGTCT

### 4.7 IAA Formation—Time Course

To test the knock‐out strains for their ability to produce auxin, a pre‐culture was prepared by inoculating 10^5^ spores of PH‐1 and PCR confirmed single and multiple knock‐out strains in 50 mL FMM and shaking the cultures at 20°C, 180 rpm for 3 days. 1 mM TAM was added to each culture, and samples were taken at time point 0 and then every 24 h over 4 days. The experiment was carried out in three replicates.

1‐mL aliquots of the cultures were transferred to 1.5‐mL tubes and centrifuged for 1 min at full speed (20,000 *g*). 500 μL of the supernatants were transferred to fresh tubes containing 500 μL methanol. The samples were vortexed for 30 s and then centrifuged at full speed for 5 min. 50 μL of these samples were transferred to HPLC vials containing 950 μL methanol resulting in a final dilution of 1:40 for the measurements. The samples were measured by LC–MS/MS as described below.

### 4.8 *AOX4* Complementation

To determine whether IAA production could be restored by insertion of a single *AOX* gene, *AOX4* was introduced into the septuple knock‐out strain. For this purpose, the *hph* cassette in pHE49 (#2212) was replaced by the *AOX4* coding region. First, primers 5′‐CTTTGACAATGCTGGTAGATG‐3′ and 5′‐ATTGTTATCACATATCGCATTAC‐3′ and genomic DNA from PH‐1 were used to amplify the *AOX4* open reading frame plus 40 bp of the flanking regions on each end. This fragment was cloned into SpeI‐SalI digested pHE29 (containing the 3′ and 5′ flanking regions and the vector backbone) by homologous recombination with the ~40 bp extension of the amplified sequence using the enzyme λ‐red‐recombinase yielding plasmid pPS27. The *nptII* cassette was cut from pII99 using BsiWI/NdeI, purified and introduced into pPS27 resulting in the plasmid pPS29, which consists of the *AOX4* coding sequence flanked by the 5′‐and the 3′‐flanking region with *nptII* adjacent. Prior to transformation of the *F. graminearum* aoxΔ^7^ strain, the plasmid was linearized using SalI. Two independent complementation transformants were obtained, termed comp.4 and comp.49 (#2970, #2971). The consequence of restoration of *AOX4* was determined by a TAM feeding assay in liquid culture. Twenty millilitres of FMM was inoculated with 10^5^ spores of selected transformants as well as the septuple knock‐out and the WT strain. After 3 days of incubation at 20°C, 180 rpm 1 mM TAM was supplemented. Samples were taken every 24 h over a total time period of 4 days starting with 0 h. For the measurements 1:40 h were prepared.

### 4.9 LC–MS/MS Determination of Auxin Metabolites

Sample analyses were carried out on an Agilent 1290 Ultra High‐Performance Liquid Chromatography system (Waldbronn) coupled to a 6500 QTrap tandem mass spectrometer (Sciex). A Hypersil Gold C18 column (50 × 2.1 mm, 1.9 μm particle size, Thermo Scientific) was used for separation. Mobile phase A consisted of 5% aqueous methanol, while mobile phase B was pure methanol. Both phases were adjusted with 0.1% formic acid (HCOOH) as well as 1 mM ammonium formate. A flow rate of 500 μL/min was used at a column temperature of 30°C and 1 μL of samples was injected. After a 0.5 min hold time of mobile phase A, a linear gradient to 100% B was used until 4.0 min. The conditions were held until 5.5 min before re‐equilibration of the column until the end of the run at 8.0 min. A diverter valve split the flow to the MS from 0.6 to 4.0 min.

Analyst 1.6.3 (Sciex) was used to control the LC–MS/MS system and to evaluate the data. A TurboV IonDrive electrospray source was operated in positive mode for ionization at 2000 V and 650°C. Ion source gases 1 and 2 were set to 60 psi, while the curtain gas was set to 35 psi. Scheduled selected monitoring reaction (sSRM) was used with a detection window of 60 s and a target scan time of 0.6 s. The used sSRM transitions can be found in Table 4.

**TABLE 4:** Used selected monitoring MS/MS transitions for auxin metabolites. Q, Quantifier; q, qualifier; Q1, selected *m/z* for quadrupole 1; Q3, selected *m/z* for quadrupole 3; R_t_, retention time (min); DP, declustering potential (V); CE, collision energy (eV); CXP, cell exit potential (V).

Compound	Q1	Q3	R_t_	DP	CE	CXP
5 OH TRP (Q)	221.0	204.1	0.97	11	15	16
5 OH TRP (q)	221.0	162.2	0.97	11	25	16
5OH IAA (Q)	192.0	146.0	1.90	6	21	18
5OH IAA (q)	192.0	90.8	1.90	6	49	12
COU‐TAM (Q)	307.0	147.0	3.18	71	23	14
COU‐TAM (q)	307.0	118.9	3.18	71	47	12
FER‐TAM (Q)	337.0	177.0	3.19	66	19	12
FER‐TAM (q)	337.0	145.0	3.19	66	41	10
I3CA POS (Q)	162.0	117.9	2.63	26	17	14
I3CA POS (q)	162.0	144.0	2.63	26	19	8
I3CHO (Q)	146.0	118.1	2.60	6	21	14
I3CHO (q)	146.0	91.0	2.60	6	35	6
IAA (Q)	176.0	130.1	2.70	31	19	8
IAA (q)	176.0	103.1	2.70	31	43	12
IAAla (Q)	247.0	130.1	2.55	6	25	14
IAAla (q)	247.0	89.9	2.55	6	15	10
IAAsp (Q)	291.0	130.1	2.28	6	27	8
IAAsp (q)	291.0	133.9	2.28	6	17	8
IA(I)leu (Q)	289.0	130.0	3.28	51	31	14
IA(I)leu (q)	289.0	86.0	3.28	51	23	10
IAPhe (Q)	323.0	130.0	3.32	46	29	14
IAPhe (q)	323.0	166.1	3.32	46	19	10
IATrp (Q)	362.0	129.9	3.21	6	49	18
IATrp (q)	362.0	188.0	3.21	6	27	10
IAVal (Q)	275.0	130.0	3.06	41	27	16
IAVal (q)	275.0	72.0	3.06	41	21	10
Indole 3 carbinol (Q)	130.0	77.0	3.66	86	37	12
Indole 3 carbinol (q)	130.0	102.9	3.66	86	29	12
Indole (Q)	118.0	91.0	2.74	6	31	14
Indole (q)	118.0	64.9	2.74	6	41	8
Serotonine (Q)	177.0	160.1	0.97	6	13	12
Serotonine (q)	177.0	115.0	0.97	6	37	14
TRamine (Q)	161.0	143.9	1.86	36	13	18
TRamine (q)	161.0	116.9	1.86	36	33	14
TRP (Q)	205.0	188.0	1.77	6	13	20
TRP (q)	205.0	145.9	1.77	6	23	14
Tryptophol (Q)	162.0	144.0	2.68	51	19	10
Tryptophol (q)	162.0	117.1	2.68	51	31	10
COF TAM (Q)	323.0	162.9	3.04	16	25	20
COF TAM (q)	323.0	161.0	3.04	16	17	10
COU SER (Q)	323.0	147.0	2.65	61	19	10
COU SER (q)	323.0	91.0	2.65	61	67	10
FER SER (Q)	353.0	177.1	2.68	56	25	14
FER SER (q)	353.0	144.9	2.68	56	41	16
IAm (Q)	175.1	77.0	2.21	31	55	10
IAm (q)	175.1	103.0	2.21	31	45	12
IAc (Q)	174.0	132.0	2.84	41	19	14
IAc (IAc)	174.0	117.0	2.84	41	37	12
IAN (Q)	157.0	130.0	2.80	21	17	6
IAN (q)	157.0	117.1	2.80	21	29	10
IBA (Q)	204.0	186.0	3.17	21	17	12
IBA (q)	204.0	130.0	3.17	21	39	8
OxIAA (Q)	192.0	146.1	2.31	16	19	10
OxIAA (q)	192.0	128.0	2.31	16	37	14
IAA Glucoside (Q)	355.1	176.0	2.30	21	17	10
IAA Glucoside (q)	355.1	130.0	2.30	21	43	14

### 4.10 Virulence Tests (Point Inoculation)

The *Fusarium* strains to be tested were sporulated in 50 mL MBS (10 g mung beans cooked in 1 L water for 20 min followed by filtration and autoclaving) by shaking at 20°C, 140 rpm for 3 days. The spores were filtered, counted and diluted to a final concentration of 4 × 10^4^ spores/mL. Four florets of two spikelets in the middle section of the ear of Apogee wheat were each inoculated with 10 μL of the spore suspension. Each ear was covered with a plastic bag that was previously sprayed with water to provide humidity and then incubated at 20°C with a day/night cycle 16 h/8 h. The progress of infection was recorded every 2 days. For these virulence tests 10 plants each were infected with either the WT strain PH‐1 or with the two isogenic aoxΔ^7^ mutants. The progress of infection was documented over 16 days. Subsequently, the whole ears were harvested and extracted for determination of DON (Steiner et al. 2020) and IAA concentrations. After homogenization of the samples, extraction was done adding 400 μL extraction solvent (acetonitrile:water:acetic acid = 79:20:1, v:v:v) per 100 mg sample. The tubes were incubated at 20°C, 180 rpm for 30 min followed by centrifugation and transfer of the supernatant to a HPLC vial.

For the determination of the changes of TRP, TAM and IAA levels over time, two spikelets were treated as described above or with sterile water as control. Ten ears per strain and time point were infected. After 3, 6, 9 and 12 days, 40 ears were harvested and extracted followed by LC–MS/MS analysis. All results were statistically tested for significance using Student's *t*‐test with cut‐offs of *p* < 0.05 and *p* < 0.01.

## Author Contributions

**Thomas Svoboda:** investigation, writing – original draft, writing – review and editing, data curation, formal analysis, methodology, visualization. **Anika Bartholomäus:** investigation, writing – review and editing. **Pia Spörhase:** investigation, writing – review and editing. **Bernhard Seidl:** investigation, writing – review and editing, data curation. **Gerlinde Wiesenberger:** investigation, supervision, writing – review and editing, project administration. **Ulrich Gueldener:** methodology, investigation, writing – review and editing. **Philipp Fruhmann:** investigation, writing – review and editing, resources. **Eduardo Beltran:** methodology, data curation, investigation, writing – review and editing. **Rudolf Krska:** supervision, funding acquisition, writing – review and editing, project administration. **Rainer Schuhmacher:** conceptualization, supervision, project administration, writing – review and editing, methodology, validation. **Asja Ceranic:** investigation, writing – review and editing, data curation. **Franz Berthiller:** investigation, supervision, writing – review and editing, project administration, methodology, conceptualization, data curation. **Gerhard Adam:** conceptualization, methodology, supervision, funding acquisition, project administration, writing – original draft, writing – review and editing, validation, investigation, visualization.

## Funding

Austrian Science fund (FWF), special research project SFB F37 Fusarium (Grant‐DOI 10.55776/F37, projects F3702, F3706 and F3715, and LAP3714 funded by DFG (Deutsche Forschungsgemeinschaft)). Development of the LC–MS/MS method was funded by the Lower Austrian government as well as European Regional Development Fund (ERDF) within the project ‘Kooperation IFA‐Tulln/IST Austria im Bereich Pflanzenhormone’ (WST3‐T‐95/020‐2013).

## Conflicts of Interest

The authors declare no conflicts of interest.

## Supporting information


**Figure S1:** Auxin biosynthesis pathways.

**Figure S2:** Hydrolysis of FER‐TAM by concentrated protein from culture supernatant. Concentrations of FER‐TAM and hydrolysis products TAM and FER in the 1/16 diluted samples. Substrate concentration in the reaction was initially 125 μM FER‐TAM.

**Figure S3:** Knock‐out scheme for the preparation of the septuple knock‐out strains (a) and PCR confirmation of the septuple knock‐out (b). Template on left from the septuple mutant DNA (strain AOXΔ7.1), right wildtype; Flanking primers in the up and downstream region of the respective gene were designed to give fragments of 1–1.6 kb. The primers A and B for each gene are indicated in Table 3. The wild‐type fragments (right, numbers indicate primer pair n of the *AOXn* gene. In wild‐type most fragments were too long for amplification in some cases unspecific second bands are generated); smaller band *aox7* mutant compared to the native gene.

**Figure S4:** Strain genealogy, short and full genotypes (strain collection numbers Adam lab).

**File S1:** Strain construction, genotypes and freezer collection numbers (Adam Lab).

**Table S1:** Expression of amine oxidases during plant infection.

## Data Availability

The data that support the findings of this study are available from the corresponding author upon reasonable request.

## References

[mpp70325-bib-0001] Aalders, T. R. , M. de Sain , F. Gawehns , et al. 2024. “Specific Members of the TOPLESS Family Are Susceptibility Genes for Fusarium Wilt in Tomato and *Arabidopsis* .” Plant Biotechnology Journal 22: 248–261. 10.1111/pbi.14183. 37822043 PMC10754003

[mpp70325-bib-0002] Back, K. 2021. “Melatonin Metabolism, Signaling and Possible Roles in Plants.” Plant Journal 105: 376–391. 10.1111/tpj.14915. 32645752

[mpp70325-bib-0003] Bassard, J.‐E. , P. Ullmann , F. Bernier , and D. Werck‐Reichhart . 2010. “Phenolamides: Bridging Polyamines to the Phenolic Metabolism.” Phytochemistry 71: 1808–1824. 10.1016/j.phytochem.2010.08.003. 20800856

[mpp70325-bib-0004] Berti, F. , E. M. Tamburello , and C. Forzato . 2025. “P‐Coumaroyl Amides From the Plant Kingdom: A Comprehensive Review of Natural Sources, Biosynthesis, and Biological Activities.” Molecules 30: 1259. 10.3390/molecules30061259. 40142036 PMC11944718

[mpp70325-bib-0005] Bethke, G. , Y. Huang , G. Hensel , et al. 2023. “UDP‐Glucosyltransferase HvUGT13248 Confers Type II Resistance to *Fusarium graminearum* in Barley.” Plant Physiology 193: 2691–2710. 10.1093/plphys/kiad467. 37610244

[mpp70325-bib-0006] Bindics, J. , M. Khan , S. Uhse , et al. 2022. “Many Ways to TOPLESS ‐ Manipulation of Plant Auxin Signalling by a Cluster of Fungal Effectors.” New Phytologist 236: 1455–1470. 10.1111/nph.18315. 35944559

[mpp70325-bib-0007] Boddu, J. , S. Cho , W. M. Kruger , and G. J. Muehlbauer . 2006. “Transcriptome Analysis of the Barley–*Fusarium graminearum* Interaction.” Molecular Plant–Microbe Interactions 19: 407–417. 10.1094/MPMI-19-0407. 16610744

[mpp70325-bib-0008] Boedi, S. , H. Berger , C. Sieber , et al. 2016. “Comparison of *Fusarium graminearum* Transcriptomes on Living or Dead Wheat Differentiates Substrate‐Responsive and Defense‐Responsive Genes.” Frontiers in Microbiology 7: 1113. 10.3389/fmicb.2016.01113. 27507961 PMC4960244

[mpp70325-bib-0009] Boutigny, A.‐L. , C. Barreau , V. Atanasova‐Penichon , M.‐N. Verdal‐Bonnin , L. Pinson‐Gadais , and F. Richard‐Forget . 2009. “Ferulic Acid, an Efficient Inhibitor of Type B Trichothecene Biosynthesis and *Tri* Gene Expression in *Fusarium* Liquid Cultures.” Mycological Research 113: 746–753. 10.1016/j.mycres.2009.02.010. 19249362

[mpp70325-bib-0010] Brown, N. A. , J. Evans , A. Mead , and K. E. Hammond‐Kosack . 2017. “A Spatial Temporal Analysis of the *Fusarium graminearum* Transcriptome During Symptomless and Symptomatic Wheat Infection.” Molecular Plant Pathology 18: 1295–1312. 10.1111/mpp.12564. 28466509 PMC5697668

[mpp70325-bib-0011] Cao, X. , H. Yang , C. Shang , S. Ma , L. Liu , and J. Cheng . 2019. “The Roles of Auxin Biosynthesis YUCCA Gene Family in Plants.” International Journal of Molecular Sciences 20: 6343. 10.3390/ijms20246343. 31888214 PMC6941117

[mpp70325-bib-0012] Ćeranić, A. , T. Svoboda , F. Berthiller , et al. 2021. “Identification and Functional Characterization of the Gene Cluster Responsible for Fusaproliferin Biosynthesis in *Fusarium proliferatum* .” Toxins 13: 468. 10.3390/toxins13070468. 34357940 PMC8310001

[mpp70325-bib-0013] Cohen, J. D. , and L. C. Strader . 2024. “An Auxin Research Odyssey: 1989–2023.” Plant Cell 36: 1410–1428. 10.1093/plcell/koae054. 38382088 PMC11062468

[mpp70325-bib-0014] Cuomo, C. A. , U. Güldener , J.‐R. Xu , et al. 2007. “The *Fusarium graminearum* Genome Reveals a Link Between Localized Polymorphism and Pathogen Specialization.” Science 317: 1400–1402. 10.1126/science.1143708. 17823352

[mpp70325-bib-0015] Denancé, N. , A. Sánchez‐Vallet , D. Goffner , and A. Molina . 2013. “Disease Resistance or Growth: The Role of Plant Hormones in Balancing Immune Responses and Fitness Costs.” Frontiers in Plant Science 4: 155. 10.3389/fpls.2013.00155. 23745126 PMC3662895

[mpp70325-bib-0016] Doppler, M. , C. Bueschl , F. Ertl , J. Woischitzschlaeger , A. Parich , and R. Schuhmacher . 2022. “Towards a Broader View of the Metabolome: Untargeted Profiling of Soluble and Bound Polyphenols in Plants.” Analytical and Bioanalytical Chemistry 414: 7421–7433. 10.1007/s00216-022-04134-z. 35678834 PMC9482910

[mpp70325-bib-0017] Doppler, M. , B. Kluger , C. Bueschl , et al. 2019. “Stable Isotope‐Assisted Plant Metabolomics: Investigation of Phenylalanine‐Related Metabolic Response in Wheat Upon Treatment With the Fusarium Virulence Factor Deoxynivalenol.” Frontiers in Plant Science 10: 1137. 10.3389/fpls.2019.01137. 31736983 PMC6831647

[mpp70325-bib-0018] Du, Y. , R. Tejos , M. Beck , et al. 2013. “Salicylic Acid Interferes With Clathrin‐Mediated Endocytic Protein Trafficking.” Proceedings of the National Academy of Sciences of the United States of America 110: 7946–7951. 10.1073/pnas.1220205110. 23613581 PMC3651428

[mpp70325-bib-0019] Fang, H. , F. Zhang , C. Zhang , et al. 2022. “Function of Hydroxycinnamoyl Transferases for the Biosynthesis of Phenolamides in Rice Resistance to *Magnaporthe oryzae* .” Journal of Genetics and Genomics 49: 776–786. 10.1016/j.jgg.2022.02.008. 35231636

[mpp70325-bib-0020] Fu, S.‐F. , J.‐Y. Wei , H.‐W. Chen , Y.‐Y. Liu , H.‐Y. Lu , and J.‐Y. Chou . 2015. “Indole‐3‐Acetic Acid: A Widespread Physiological Code in Interactions of Fungi With Other Organisms.” Plant Signaling & Behavior 10: e1048052. 10.1080/15592324.2015.1048052. 26179718 PMC4623019

[mpp70325-bib-0021] Gardiner, D. M. , K. Kazan , S. Praud , F. J. Torney , A. Rusu , and J. M. Manners . 2010. “Early Activation of Wheat Polyamine Biosynthesis During Fusarium Head Blight Implicates Putrescine as an Inducer of Trichothecene Mycotoxin Production.” BMC Plant Biology 10: 289. 10.1186/1471-2229-10-289. 21192794 PMC3022911

[mpp70325-bib-0022] Gomes, G. L. B. , and K. C. Scortecci . 2021. “Auxin and Its Role in Plant Development: Structure, Signalling, Regulation and Response Mechanisms.” Plant Biology 23: 894–904. 10.1111/plb.13303. 34396657

[mpp70325-bib-0023] Gunnaiah, R. , and A. C. Kushalappa . 2014. “Metabolomics Deciphers the Host Resistance Mechanisms in Wheat Cultivar Sumai‐3, Against Trichothecene Producing and Non‐Producing Isolates of *Fusarium graminearum* .” Plant Physiology and Biochemistry: PPB 83: 40–50. 10.1016/j.plaphy.2014.07.002. 25084325

[mpp70325-bib-0024] Gunnaiah, R. , A. C. Kushalappa , R. Duggavathi , S. Fox , and D. J. Somers . 2012. “Integrated Metabolo‐Proteomic Approach to Decipher the Mechanisms by Which Wheat QTL (*Fhb1*) Contributes to Resistance Against *Fusarium graminearum* .” PLoS One 7: e40695. 10.1371/journal.pone.0040695. 22866179 PMC3398977

[mpp70325-bib-0025] Hao, Q. , W. Wang , X. Han , et al. 2018. “Isochorismate‐Based Salicylic Acid Biosynthesis Confers Basal Resistance to *Fusarium graminearum* in Barley.” Molecular Plant Pathology 19: 1995–2010. 10.1111/mpp.12675. 29517854 PMC6638154

[mpp70325-bib-0026] Hayashi, K.‐I. , K. Arai , Y. Aoi , et al. 2021. “The Main Oxidative Inactivation Pathway of the Plant Hormone Auxin.” Nature Communications 12: 6752. 10.1038/s41467-021-27020-1. PMC8608799 34811366

[mpp70325-bib-0027] Hu, S. , H. Zhao , Q. Wu , et al. 2025. “A Putative Auxin Efflux Carrier FgAec1 Contributes to the Pathogenicity of *Fusarium graminearum* .” BMC Biology 23: 329. 10.1186/s12915-025-02438-x. 41174574 PMC12577076

[mpp70325-bib-0028] Jansen, C. , D. von Wettstein , W. Schäfer , K.‐H. Kogel , A. Felk , and F. J. Maier . 2005. “Infection Patterns in Barley and Wheat Spikes Inoculated With Wild‐Type and Trichodiene Synthase Gene Disrupted *Fusarium graminearum* .” Proceedings of the National Academy of Sciences of the United States of America 102: 16892–16897. 10.1073/pnas.0508467102. 16263921 PMC1283850

[mpp70325-bib-0029] Jin, S. , and M. Yoshida . 2000. “Antifungal Compound, Feruloylagmatine, Induced in Winter Wheat Exposed to a Low Temperature.” Bioscience, Biotechnology, and Biochemistry 64: 1614–1617. 10.1271/bbb.64.1614. 10993146

[mpp70325-bib-0030] Kanjanaphachoat, P. , B.‐Y. Wei , S.‐F. Lo , et al. 2012. “Serotonin Accumulation in Transgenic Rice by Over‐Expressing Tryptophan Decarboxlyase Results in a Dark Brown Phenotype and Stunted Growth.” Plant Molecular Biology 78: 525–543. 10.1007/s11103-012-9882-5. 22297847

[mpp70325-bib-0031] Karre, S. , A. Kumar , K. Yogendra , U. Kage , A. Kushalappa , and J.‐B. Charron . 2019. “HvWRKY23 Regulates Flavonoid Glycoside and Hydroxycinnamic Acid Amide Biosynthetic Genes in Barley to Combat Fusarium Head Blight.” Plant Molecular Biology 100: 591–605. 10.1007/s11103-019-00882-2. 31098785

[mpp70325-bib-0032] Kidd, B. N. , N. Y. Kadoo , B. Dombrecht , et al. 2011. “Auxin Signaling and Transport Promote Susceptibility to the Root‐Infecting Fungal Pathogen *Fusarium oxysporum* in *Arabidopsis* .” Molecular Plant–Microbe Interactions 24: 733–748. 10.1094/MPMI-08-10-0194. 21281113

[mpp70325-bib-0033] Kovalsky Paris, M. P. 2013. “Functional Characterization of Plant UDP‐Glucosyltransferases for Their Ability to Conjugate Fusarium Secondary Metabolites.” [PhD thesis]. Universität für Bodenkultur Wien. https://permalink.obvsg.at/bok/AC10775161.

[mpp70325-bib-0034] Kristensen, B. K. , K. Burhenne , and S. K. Rasmussen . 2004. “Peroxidases and the Metabolism of Hydroxycinnamic Acid Amides in Poaceae.” Phytochemistry Reviews 3: 127–140. 10.1023/B:PHYT.0000047800.59980.6e.

[mpp70325-bib-0035] Kunkel, B. N. , and C. P. Harper . 2018. “The Roles of Auxin During Interactions Between Bacterial Plant Pathogens and Their Hosts.” Journal of Experimental Botany 69: 245–254. 10.1093/jxb/erx447. 29272462

[mpp70325-bib-0036] Kushalappa, A. C. , K. N. Yogendra , and S. Karre . 2016. “Plant Innate Immune Response: Qualitative and Quantitative Resistance.” Critical Reviews in Plant Sciences 35: 38–55. 10.1080/07352689.2016.1148980.

[mpp70325-bib-0037] Liao, C.‐J. , S. Hailemariam , A. Sharon , and T. Mengiste . 2022. “Pathogenic Strategies and Immune Mechanisms to Necrotrophs: Differences and Similarities to Biotrophs and Hemibiotrophs.” Current Opinion in Plant Biology 69: 102291. 10.1016/j.pbi.2022.102291. 36063637

[mpp70325-bib-0038] Liu, S. , J. Jiang , Z. Ma , et al. 2022. “The Role of Hydroxycinnamic Acid Amide Pathway in Plant Immunity.” Frontiers in Plant Science 13: 922119. 10.3389/fpls.2022.922119. 35812905 PMC9257175

[mpp70325-bib-0039] Lowe, R. , M. Jubault , G. Canning , M. Urban , and K. E. Hammond‐Kosack . 2012. “The Induction of Mycotoxins by Trichothecene Producing *Fusarium* Species.” In Plant Fungal Pathogens: Methods and Protocols, edited by M. D. Bolton and B. P. H. J. Thomma , 439–455. Humana Press. 10.1007/978-1-61779-501-5_27 22183670

[mpp70325-bib-0040] Lowe, R. G. T. , O. McCorkelle , M. Bleackley , et al. 2015. “Extracellular Peptidases of the Cereal Pathogen *Fusarium graminearum* .” Frontiers in Plant Science 6: 962. 10.3389/fpls.2015.00962. 26635820 PMC4645717

[mpp70325-bib-0041] Luo, K. , H. Rocheleau , P.‐F. Qi , Y.‐L. Zheng , H.‐Y. Zhao , and T. Ouellet . 2016. “Indole‐3‐Acetic Acid in *Fusarium graminearum*: Identification of Biosynthetic Pathways and Characterization of Physiological Effects.” Fungal Biology 120: 1135–1145. 10.1016/j.funbio.2016.06.002. 27567719

[mpp70325-bib-0042] Luo, P. , D. Di , L. Wu , J. Yang , Y. Lu , and W. Shi . 2022. “MicroRNAs Are Involved in Regulating Plant Development and Stress Response Through Fine‐Tuning of TIR1/AFB‐Dependent Auxin Signaling.” International Journal of Molecular Sciences 23: 510. 10.3390/ijms23010510. 35008937 PMC8745101

[mpp70325-bib-0043] Luo, P. , T.‐T. Li , W.‐M. Shi , Q. Ma , and D.‐W. Di . 2023. “The Roles of GRETCHEN HAGEN3 (GH3)‐Dependent Auxin Conjugation in the Regulation of Plant Development and Stress Adaptation.” Plants 12: 4111. 10.3390/plants12244111. 38140438 PMC10747189

[mpp70325-bib-0044] Mackintosh, C. A. , D. F. Garvin , L. E. Radmer , S. J. Heinen , and G. J. Muehlbauer . 2006. “A Model Wheat Cultivar for Transformation to Improve Resistance to Fusarium Head Blight.” Plant Cell Reports 25: 313–319. 10.1007/s00299-005-0059-4. 16252090

[mpp70325-bib-0045] Macoy, D. M. , W.‐Y. Kim , S. Y. Lee , and M. G. Kim . 2015a. “Biosynthesis, Physiology, and Functions of Hydroxycinnamic Acid Amides in Plants.” Plant Biotechnology Reports 9: 269–278. 10.1007/s11816-015-0368-1.

[mpp70325-bib-0046] Macoy, D. M. , W.‐Y. Kim , S. Y. Lee , and M. G. Kim . 2015b. “Biotic Stress Related Functions of Hydroxycinnamic Acid Amide in Plants.” Journal of Plant Biology 58: 156–163. 10.1007/s12374-015-0104-y.

[mpp70325-bib-0047] Macoy, D. M. J. , S. Uddin , G. Ahn , et al. 2022. “Effect of Hydroxycinnamic Acid Amides, Coumaroyl Tyramine and Coumaroyl Tryptamine on Biotic Stress Response in *Arabidopsis* .” Journal of Plant Biology 65: 145–155. 10.1007/s12374-021-09341-2.

[mpp70325-bib-0048] Makandar, R. , V. J. Nalam , H. Lee , H. N. Trick , Y. Dong , and J. Shah . 2012. “Salicylic Acid Regulates Basal Resistance to Fusarium Head Blight in Wheat.” Molecular Plant–Microbe Interactions 25: 431–439. 10.1094/MPMI-09-11-0232. 22112217

[mpp70325-bib-0049] Mano, Y. , and K. Nemoto . 2012. “The Pathway of Auxin Biosynthesis in Plants.” Journal of Experimental Botany 63: 2853–2872. 10.1093/jxb/ers091. 22447967

[mpp70325-bib-0050] Navarrete, F. , M. Gallei , A. E. Kornienko , et al. 2022. “TOPLESS Promotes Plant Immunity by Repressing Auxin Signaling and Is Targeted by the Fungal Effector Naked1.” Plant Communications 3: 100269. 10.1016/j.xplc.2021.100269. 35529945 PMC9073326

[mpp70325-bib-0051] Niehaus, E.‐M. , M. Münsterkötter , R. H. Proctor , et al. 2016. “Comparative “Omics” of the *Fusarium fujikuroi* Species Complex Highlights Differences in Genetic Potential and Metabolite Synthesis.” Genome Biology and Evolution 8: 3574–3599. 10.1093/gbe/evw259. 28040774 PMC5203792

[mpp70325-bib-0052] Park, H. L. , Y. Yoo , T.‐R. Hahn , S. H. Bhoo , S.‐W. Lee , and M.‐H. Cho . 2014. “Antimicrobial Activity of UV‐Induced Phenylamides From Rice Leaves.” Molecules 19: 18139–18151. 10.3390/molecules191118139. 25383752 PMC6271653

[mpp70325-bib-0053] Peng, M. , Y. Gao , W. Chen , et al. 2016. “Evolutionarily Distinct BAHD N‐Acyltransferases Are Responsible for Natural Variation of Aromatic Amine Conjugates in Rice.” Plant Cell 28: 1533–1550. 10.1105/tpc.16.00265. 27354554 PMC4981135

[mpp70325-bib-0054] Petti, C. , K. Reiber , S. S. Ali , M. Berney , and F. M. Doohan . 2012. “Auxin as a Player in the Biocontrol of Fusarium Head Blight Disease of Barley and Its Potential as a Disease Control Agent.” BMC Plant Biology 12: 224. 10.1186/1471-2229-12-224. 23173736 PMC3556313

[mpp70325-bib-0055] Qi, P.‐F. , Y.‐Z. Zhang , C.‐H. Liu , et al. 2019. “Functional Analysis of FgNahG Clarifies the Contribution of Salicylic Acid to Wheat ( *Triticum aestivum* ) Resistance Against Fusarium Head Blight.” Toxins 11: 59. 10.3390/toxins11020059. 30678154 PMC6410203

[mpp70325-bib-0056] Rosazza, J. P. , Z. Huang , L. Dostal , T. Volm , and B. Rousseau . 1995. “Review: Biocatalytic Transformations of Ferulic Acid: An Abundant Aromatic Natural Product.” Journal of Industrial Microbiology 15: 457–471. 10.1007/BF01570016. 8821508

[mpp70325-bib-0057] Rosquete, M. R. , E. Barbez , and J. Kleine‐Vehn . 2012. “Cellular Auxin Homeostasis: Gatekeeping Is Housekeeping.” Molecular Plant 5: 772–786. 10.1093/mp/ssr109. 22199236

[mpp70325-bib-0058] Roumani, M. , S. Besseau , D. Gagneul , C. Robin , and R. Larbat . 2021. “Phenolamides in Plants: An Update on Their Function, Regulation, and Origin of Their Biosynthetic Enzymes.” Journal of Experimental Botany 72: 2334–2355. 10.1093/jxb/eraa582. 33315095

[mpp70325-bib-0059] Sardar, P. , and F. Kempken . 2018. “Characterization of Indole‐3‐Pyruvic Acid Pathway‐Mediated Biosynthesis of Auxin in *Neurospora crassa* .” PLoS One 13: e0192293. 10.1371/journal.pone.0192293. 29420579 PMC5805262

[mpp70325-bib-0060] Schweiger, W. , B. Steiner , S. Vautrin , et al. 2016. “Suppressed Recombination and Unique Candidate Genes in the Divergent Haplotype Encoding Fhb1, a Major Fusarium Head Blight Resistance Locus in Wheat.” Theoretical and Applied Genetics 129: 1607–1623. 10.1007/s00122-016-2727-x. 27174222 PMC4943984

[mpp70325-bib-0061] Sevinç, E. , N. Özer , and N. Desen Köycü . 2025. “Reactions of Some Summer Pumpkin Cultivars to Root and Crown Rot Caused by Soil Infestation With *Fusarium proliferatum*: Assessment Based on Disease Severity, Growth Parameters and Phenolic Compounds.” Journal of Crop Health 77: 137. 10.1007/s10343-025-01203-y.

[mpp70325-bib-0062] Shang, H. , B. Ji , T. Ouellet , et al. 2026. “Auxin Accumulation in Cereals After Infection by *Fusarium graminearum*: Putative Biosynthetic Pathways and Preferences.” Stress Biology 6: 10. 10.1007/s44154-025-00267-0. 41609985 PMC12855686

[mpp70325-bib-0063] Song, T. , Q. Huo , C. Li , et al. 2024. “The Biosynthesis of Storage Reserves and Auxin Is Coordinated by a Hierarchical Regulatory Network in Maize Endosperm.” New Phytologist 243: 1855–1869. 10.1111/nph.19949. 38962989

[mpp70325-bib-0064] Spörhase, P. 2015. “*Fusarium graminearum* Can Subvert the Synthesis of Tryptamine‐Derived Defense Compounds to Produce Auxin.” [Thesis]. Universität für Bodenkultur Wien. https://permalink.obvsg.at/bok/AC13096363.

[mpp70325-bib-0065] Steiner, D. , M. Sulyok , A. Malachová , A. Mueller , and R. Krska . 2020. “Realizing the Simultaneous Liquid Chromatography‐Tandem Mass Spectrometry Based Quantification of >1200 Biotoxins, Pesticides and Veterinary Drugs in Complex Feed.” Journal of Chromatography. A 1629: 461502. 10.1016/j.chroma.2020.461502. 32841773

[mpp70325-bib-0066] Su, P. , L. Zhao , W. Li , et al. 2021. “Integrated Metabolo‐Transcriptomics and Functional Characterization Reveals That the Wheat Auxin Receptor TIR1 Negatively Regulates Defense Against *Fusarium graminearum* .” Journal of Integrative Plant Biology 63: 340–352. 10.1111/jipb.12992. 32678930

[mpp70325-bib-0067] Sugawara, S. , S. Hishiyama , Y. Jikumaru , et al. 2009. “Biochemical Analyses of Indole‐3‐Acetaldoxime‐Dependent Auxin Biosynthesis in *Arabidopsis* .” Proceedings of the National Academy of Sciences of the United States of America 106: 5430–5435. 10.1073/pnas.0811226106. 19279202 PMC2664063

[mpp70325-bib-0068] Takao, K. , K. Toda , T. Saito , and Y. Sugita . 2017. “Synthesis of Amide and Ester Derivatives of Cinnamic Acid and Its Analogs: Evaluation of Their Free Radical Scavenging and Monoamine Oxidase and Cholinesterase Inhibitory Activities.” Chemical and Pharmaceutical Bulletin 65: 1020–1027. 10.1248/cpb.c17-00416. 29093288

[mpp70325-bib-0069] Tan, S. , M. Abas , I. Verstraeten , et al. 2020. “Salicylic Acid Targets Protein Phosphatase 2A to Attenuate Growth in Plants.” Current Biology: CB 30: 381–395. 10.1016/j.cub.2019.11.058. 31956021 PMC6997888

[mpp70325-bib-0070] Tanaka, E. , C. Tanaka , N. Mori , Y. Kuwahara , and M. Tsuda . 2003. “Phenylpropanoid Amides of Serotonin Accumulate in Witches' Broom Diseased Bamboo.” Phytochemistry 64: 965–969. 10.1016/s0031-9422(03)00429-1. 14561512

[mpp70325-bib-0071] Tararina, M. A. , and K. N. Allen . 2020. “Bioinformatic Analysis of the Flavin‐Dependent Amine Oxidase Superfamily: Adaptations for Substrate Specificity and Catalytic Diversity.” Journal of Molecular Biology 432: 3269–3288. 10.1016/j.jmb.2020.03.007. 32198115 PMC7321841

[mpp70325-bib-0072] Tivendale, N. D. , N. W. Davies , P. P. Molesworth , et al. 2010. “Reassessing the Role of *N*‐Hydroxytryptamine in Auxin Biosynthesis.” Plant Physiology 154: 1957–1965. 10.1104/pp.110.165803. 20974893 PMC2996026

[mpp70325-bib-0073] Tsavkelova, E. , B. Oeser , L. Oren‐Young , et al. 2012. “Identification and Functional Characterization of Indole‐3‐Acetamide‐Mediated IAA Biosynthesis in Plant‐Associated *Fusarium* Species.” Fungal Genetics and Biology 49: 48–57. 10.1016/j.fgb.2011.10.005. 22079545

[mpp70325-bib-0074] Twaruschek, K. , P. Spörhase , H. Michlmayr , G. Wiesenberger , and G. Adam . 2018. “New Plasmids for *Fusarium* Transformation Allowing Positive‐Negative Selection and Efficient Cre‐loxP Mediated Marker Recycling.” Frontiers in Microbiology 9: 1954. 10.3389/fmicb.2018.01954. 30258410 PMC6143793

[mpp70325-bib-0075] Waadt, R. , C. A. Seller , P.‐K. Hsu , Y. Takahashi , S. Munemasa , and J. I. Schroeder . 2022. “Plant Hormone Regulation of Abiotic Stress Responses.” Nature Reviews Molecular Cell Biology 23: 680–694. 10.1038/s41580-022-00479-6. 35513717 PMC9592120

[mpp70325-bib-0076] Wang, L. , Q. Li , Z. Liu , et al. 2018. “Integrated Transcriptome and Hormone Profiling Highlight the Role of Multiple Phytohormone Pathways in Wheat Resistance Against Fusarium Head Blight.” PLoS One 13: e0207036. 10.1371/journal.pone.0207036. 30403737 PMC6221353

[mpp70325-bib-0077] Wang, Q.‐J. , X. Gao , H. Gong , X.‐R. Lin , D. Saint‐Leger , and J. Senee . 2011. “Chemical Stability and Degradation Mechanisms of Ferulic Acid (F.A) Within Various Cosmetic Formulations.” Journal of Cosmetic Science 62: 483–503. 22152493

[mpp70325-bib-0078] Xue, R. , N. Gao , J. Chen , et al. 2025. “Hydroxycinnamic Acid Amides in Rice: Biosynthesis, Distribution, Function, and Implication for Crop Development.” Frontiers in Plant Science 16: 1525268. 10.3389/fpls.2025.1525268. 40510177 PMC12158696

[mpp70325-bib-0079] Zeiss, D. R. , L. A. Piater , and I. A. Dubery . 2021. “Hydroxycinnamate Amides: Intriguing Conjugates of Plant Protective Metabolites.” Trends in Plant Science 26: 184–195. 10.1016/j.tplants.2020.09.011. 33036915

[mpp70325-bib-0080] Zhao, Y. 2010. “Auxin Biosynthesis and Its Role in Plant Development.” Annual Review of Plant Biology 61: 49–64. 10.1146/annurev-arplant-042809-112308. PMC3070418 20192736

[mpp70325-bib-0081] Zhao, Y. , S. K. Christensen , C. Fankhauser , et al. 2001. “A Role for Flavin Monooxygenase‐Like Enzymes in Auxin Biosynthesis.” Science 291: 306–309. 10.1126/science.291.5502.306. 11209081

