# Supplementary material for: The Fungal Pathogen Fusarium graminearum Can Hydrolyse Tryptamine‐Derived Hydroxy‐Cinnamic Acid Amide Defence Compounds, and Generates Highly Increased Auxin Levels in Wheat via Copper Amine‐Oxidases
Source: Mol Plant Pathol. 2026 Aug 19;27(8):e70325. doi: 10.1111/mpp.70325 (PMC13490858; doi:10.1111/mpp.70325)
Supplement: Supplementary file 1 [file MPP-27-e70325-s003.pdf]

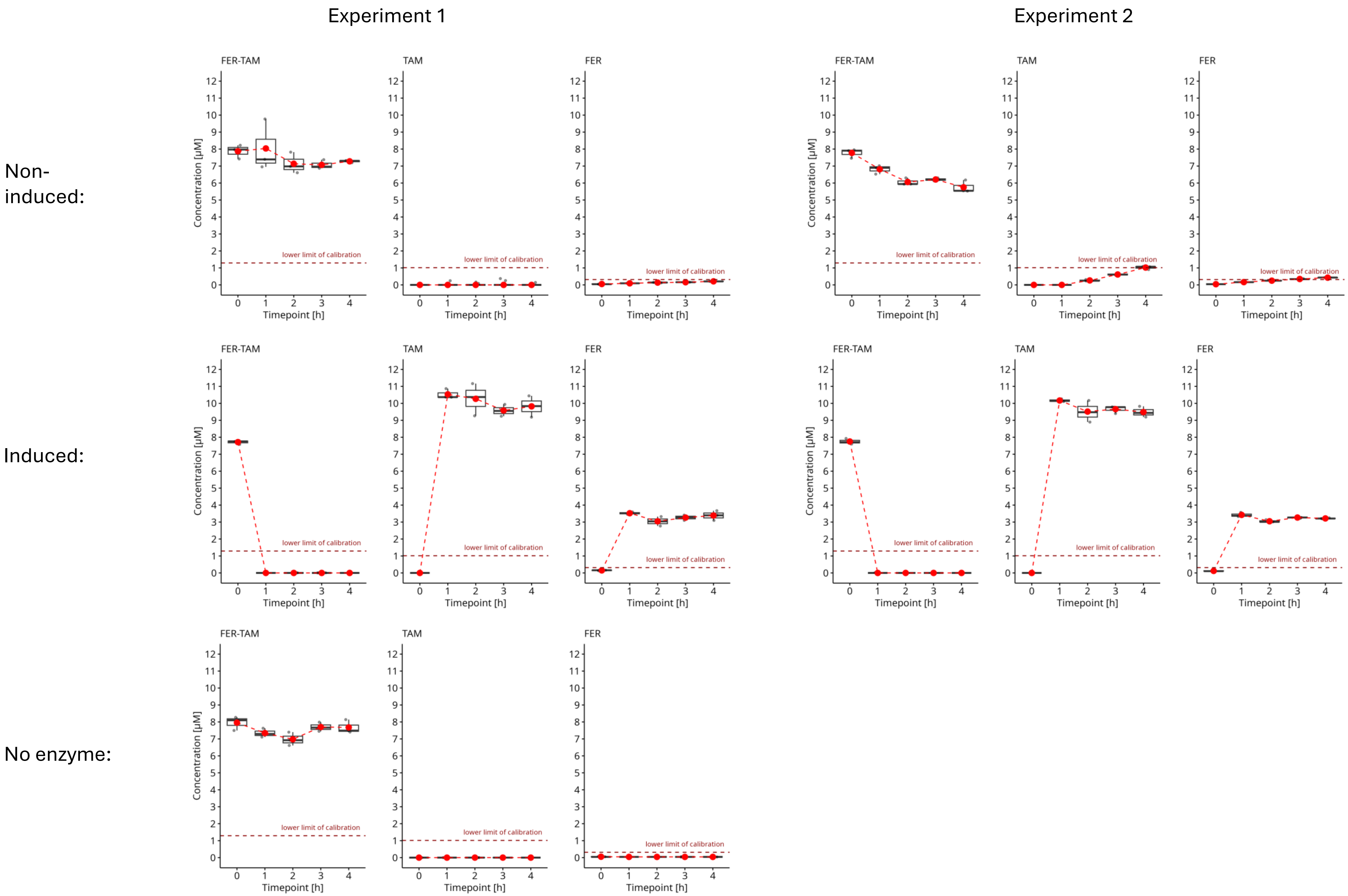

Experiment 1
Experiment 2
Non-induced:
Induced:
No enzyme:
FER-TAM
TAM
FER
Concentration [µM]
Timepoint [h]
lower limit of calibration
