# Supplementary material for: The Fungal Pathogen Fusarium graminearum Can Hydrolyse Tryptamine‐Derived Hydroxy‐Cinnamic Acid Amide Defence Compounds, and Generates Highly Increased Auxin Levels in Wheat via Copper Amine‐Oxidases
Source: Mol Plant Pathol. 2026 Aug 19;27(8):e70325. doi: 10.1111/mpp.70325 (PMC13490858; doi:10.1111/mpp.70325)
Supplement: Supplementary file 2 [file MPP-27-e70325-s006.pdf]

Figure S1:

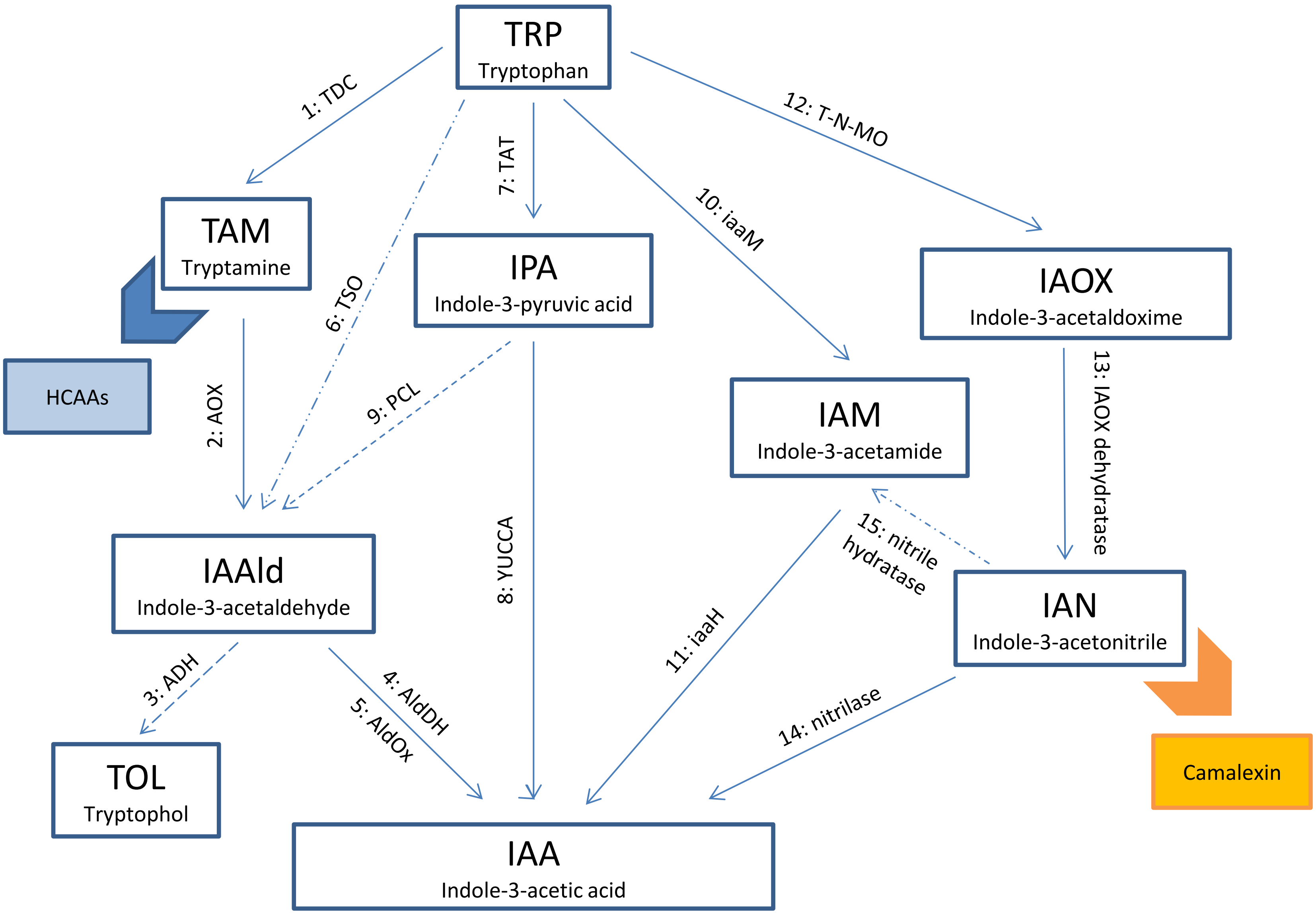

TRP
Tryptophan
1: TDC
7: TAT
10: iaaM
12: T-N-MO
6: TSO
TAM
Tryptamine
IPA
Indole-3-pyruvic acid
IAOX
Indole-3-acetaldoxime
HCAAs
2: AOX
9: PCL
IAM
Indole-3-acetamide
13: IAOX dehydratase
8: YUCCA
15: nitrile hydratase
IAAld
Indole-3-acetaldehyde
IAN
Indole-3-acetonitrile
3: ADH
4: AldDH
5: AldOx
11: iaaH
14: nitrilase
Camalexin
TOL
Tryptophol
IAA
Indole-3-acetic acid

Figure S1: Biochemical pathways from tryptophan to auxin (IAA)

Co-substrates and byproducts are not shown (for structures of compounds and details see KEGG or Metacyc reaction links (R and RXN numbers below). Rare or less relevant pathways are indicated by dashed lines. A hypothesis is that the pathogen may convert intermediates of abundant defense compounds into defense suppressing auxin (HCAAs in case of grasses, or the phytoalexin precurser IAN in Arabidopsis).

1:TDC - Tryptophan/aromatic aminoacid decarboxylase (L-Tryptophan decarboxy-lyase): R00685.

2: AOX - Amine oxidase (Tryptamine:oxygen oxidoreductase (deaminating): R02173.

3: ADH - Alcohol dehydrogenase (indole-3-ethanol:NAD+ oxidoreductase): R02679

4: AldDH (dehydrogenase) - Indole-3-acetaldehyde:NAD+ oxidoreductase: R02678

5: AldOX - indole-3-acetaldehyde oxidase (indole-3-acetaldehyde:oxygen oxidoreductase): R02681

6: TSO - tryptophan side chain oxidase (tryptophan 2'-dioxygenase) – restricted to *Pseudomonas*?: RXN-5402

7: TAT - tryptophan aminotransferase (L-Tryptophan:2-oxoglutarate aminotransferase): R00684

8: YUCCA - indole-3-pyruvate monooxygenase R10181

9: PCL - Pyruvate/3-(indol-3-yl)pyruvate carboxy-lyase R01974

10: iaaM - tryptophan 2-monooxygenase (L-tryptophan:oxygen 2-oxidoreductase (decarboxylating) R00679

11: iaaH - Indole-3-acetamide amidohydrolase: R03096

12: T-NMO - tryptophan N-monooxygenase (L-tryptophan,NADPH:oxygen oxidoreductase (N-hydroxylating, decarboxylating): R08160

13: IAOX-Dehydratase - indoleacetaldoxime dehydratase/ 3-Indoleacetaldoxime hydro-lyase: R04093

14: Nitrilase - Indoleacetonitrile aminohydrolase: R03093

15: Nitrile hydratase - Indole-3-acetamide hydro-lyase: R04020

16: Myrosinase + EPS (Epithiospecifier protein) - e.g. with Glucobrassicin: R04094
