# Supplementary material for: The Fungal Pathogen Fusarium graminearum Can Hydrolyse Tryptamine‐Derived Hydroxy‐Cinnamic Acid Amide Defence Compounds, and Generates Highly Increased Auxin Levels in Wheat via Copper Amine‐Oxidases
Source: Mol Plant Pathol. 2026 Aug 19;27(8):e70325. doi: 10.1111/mpp.70325 (PMC13490858; doi:10.1111/mpp.70325)
Supplement: Supplementary file 3 [file MPP-27-e70325-s002.docx]

**Supplementary Table 1:** Expression of amine oxidases during plant infection

The RNASeq experiment has been described in (<https://pubmed.ncbi.nlm.nih.gov/27174222/> ). Sampling of mRNAs was done after 3, 6, 12, 24,26 and 48 hours after infection with F. graminearum. The data sets are available in the EBI ArrayExpress ([http://www.ebi.ac.uk/arrayexpress/](http://www.ebi.ac.uk/arrayexpress/)) repository under the accession number E-MTAB-4222. (No reads of the fungal genes were observed in the mock treatment). For comparison also an early auxin responsive gene (SAUR: small auxin upregulated is indicated).

Data were extracted from <https://pgsb.helmholtz-muenchen.de/cgi-bin/db2/BOKUnils/index.cgi>, accessed 2016‑03-20, now defunct.

| Sequence ID | Description | Fg_03h | Fg_06h | Fg_12h | Fg_24h | Fg_36h | Fg_48h |
| --- | --- | --- | --- | --- | --- | --- | --- |
| FGSG_15961 | AOX1 | 0 | 33.6 | 66.8 | 298.7 | 197.6 | 135.3 |
| FGSG_03278 | AOX2 | 0 | 0 | 41.7 | 316.7 | 272.7 | 203.1 |
| FGSG_12129 | AOX3 | 0 | 0 | 6.6 | 100.8 | 73 | 56.6 |
| FGSG_02279 | AOX4 | 0 | 0 | 0 | 109.1 | 47 | 38.8 |
| FGSG_01761 | AOX5Ψ | 0 | 0 | 0 | 0 | 0 | 0 |
| FGSG_10587 | AOX6 | 0 | 0 | 0 | 0 | 0 | 54.4 |
| FGSG_10677 | AOX7 | 0 | 0 | 0 | 28.2 | 51.8 | 56.1 |
| FGSG_13604 | AOX8 | 0 | 0 | 0 | 0 | 6.3 | 0.9 |
|  |  |  |  |  |  |  |  |
| FGSG_03537 | TRI5 | 0 | 0 | 0 | 26.7 | 780.2 | 1001.1 |
|  |  |  |  |  |  |  |  |
| Traes_7DL_97DA08203 | SAUR-like auxin-resp. | 1,6 | 2,9 | 11 | 18.9 | 13.2 | 32.9 |
